# Mitogenomic phylogeny of nymphalid subfamilies confirms the basal clade position of Danainae (Insecta: Lepidoptera: Nymphalidae)

**DOI:** 10.1002/ece3.10263

**Published:** 2023-07-14

**Authors:** Muhammad Asghar Hassan, Rongrong Shen, Lan Zhang, Taslima Sheikh, Jichun Xing

**Affiliations:** ^1^ The Provincial Special Key Laboratory for Development and Utilization of Insect Resources, Institute of Entomology Guizhou University Guiyang China; ^2^ Department of Biological Sciences University of Memphis Memphis Tennessee USA; ^3^ Department of Zoology Sunrise University Alwar Alwar India

**Keywords:** brush‐footed butterflies, common jesters, mitochondrial genome, Papilionoidea, systematics

## Abstract

The phylogenetic relationships among the nymphalid subfamilies have largely been resolved using both morphological and molecular datasets, with the exception of a conflicting basal clade position for Libytheinae or Danainae that remains contentious between morphological and molecular studies. Several phylogenomic analyses have found that the danaine clade is sister to other nymphalid subfamilies; however, it largely depends on utilizing different molecular datasets, analysis methods, and taxon sampling. This study aimed to resolve the basal clade position and relationships among subfamilies and tribes of Nymphalinae by combining the most comprehensive available mitogenomic datasets with various analyses methods by incorporating a new *Symbrenthia lilaea* Hewitson sequence data. Phylogenetic relationships among 11 nymphalid subfamilies and the tribes of Nymphalinae were inferred by combining new and available mitogenomic sequence data from 80 ingroup and six outgroup species. The phylogenetic trees were reconstructed using maximum‐likelihood (ML) and Bayesian inference (BI) methods based on five concatenated datasets: amino acid sequences and nucleotides from different combinations of protein‐coding genes (PCGs), ribosomal RNA (rRNAs), and transfer RNA (tRNAs). Danainae is well‐supported as the basal clade and sister to the remaining nymphalid subfamilies, except for the paraphyletic Libytheinae. Libytheinae was either recovered as a sister to the danaine clade followed by the satyrine clade or sister to the nymphaline + heliconiine clades, and is consistent with recent phylogenetic studies on Nymphalidae. The monophyletic Nymphalinae has been recovered in all analyses and resolves tribal‐level relationships with high support values in both BI and ML analyses. We supported the monophyletic Nymphalini as a sister clade to Victorini, Melitaeini, and Kallimini + Junoniini with high supporting values in BI and ML analyses, which is consistent with previously published morphological and molecular studies.

## Graphical abstract

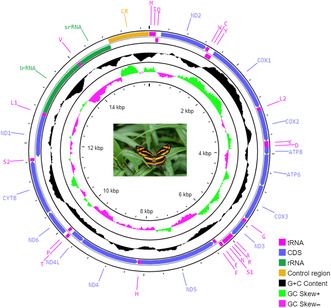

## 1 INTRODUCTION

Nymphalidae is the largest butterfly family with 12 extant subfamilies, 40 tribes, 559 genera, and ~6400 described species distributed worldwide: Danainae, Calinaginae, Charaxinae, Satyrinae, Libytheinae, Pseudergolinae, Apaturinae, Biblidinae, Cyrestinae, Nymphalinae, Heliconiinae, and Limenitidinae (Chazot et al., 2021; Wahlberg et al., 2009). Nymphalid butterflies, which likely originated in the Oriental region and repeatedly dispersed throughout the rest of the world during the early Eocene, diverged from its sister clade around 90 mya ago in the Late Cretaceous, and started to diversify around 84.6 mya ago (Chazot et al., 2021). The taxonomic levels and phylogenetic relationships among different subfamilies have mainly been resolved based on both morphological and molecular datasets. However, the basal clade position is still unresolved: either Danainae based on molecular datasets (Chazot et al., 2021; Hao et al., 2013; Shi et al., 2015; Wu et al., 2014), or Libytheinae (Espeland et al., 2018; Freitas & Brown, 2004; Wahlberg et al., 2003, 2009) based on molecular datasets, adult morphology, and similarities in the immatures with Pieridae, a hypothesis proposed 65 years ago (previously treated as distinct family; Ehrlich, 1958), has been suggested as the sister to all Nymphalidae. However, recent mitogenomic and morphological studies support Danainae as the basal clade of Nymphalidae, whereas the paraphyletic Libytheinae remains a mystery (Hao et al., 2013; Shi et al., 2015; Wu et al., 2014). Kawahara (2009) published the landmark contribution on the cladistic analyses of both extant and extinct species of Libytheinae to date, but the complete mitochondrial genome data of libytheine species are scarce and must be updated to understand its paraphyletic relationship among other nymphalid subfamilies.

The subfamily Nymphalinae comprises five tribes, Nymphalini, Kallimini, Victorinini, Junoniini, Melitaeini, and probably the Coeini (Su et al., 2017; Wahlberg et al., 2005). Nymphalinae has been recovered as a monophyletic clade and strongly supported in both morphological and mitogenomic studies (Shi et al., 2015; Su et al., 2017; Wahlberg et al., 2003, 2005; Wahlberg & Wheat, 2008; Wu et al., 2014); however, the tribe‐level relationships are mainly dependent on the use of different molecular datasets, analyses methods, and taxon sampling (Nylin et al., 2001; Su et al., 2017; Wu et al., 2014). Wahlberg et al. (2005) supported the monophyletic Nymphalinae with Nymphalini as a basal clade and sister to Victorini, Melitaeini, and Kallimini + Junoniini based on both morphological and molecular datasets. However, the Kallimini + Junoniini clade does not support recent mitogenomic studies, which either recovered Kallimini as sister to Junoniini + Melitaeini (Wu et al., 2014) or sister to the clade comprising Melitaeini and Junoniini (Shi et al., 2015), or paraphyletic Kallimini (Kim et al., 2021). These findings might be due to taxon sampling biases; however, the mitogenomic data on Nymphalidae has significantly increased in the last two decades and are available on NCBI.

This study aims to reconstruct the phylogenetic relationships among the nymphalid subfamilies and tribes of Nymphalinae by using the most comprehensive mitogenomic datasets available on Nymphalidae. Here, we included 80 ingroup species in 11 subfamilies that recovered consistent topologies and supported Danainae as a basal clade to all subfamilies. The subfamily Nymphalinae also appeared as monophyletic and Nymphalini as a basal clade and is sister to Victorini, Melitaeini, and Kallimini + Junoniini.

## 2 MATERIALS AND METHODS

### 2.1 Taxon sampling, DNA extraction, and sequencing

**FIGURE 1:**
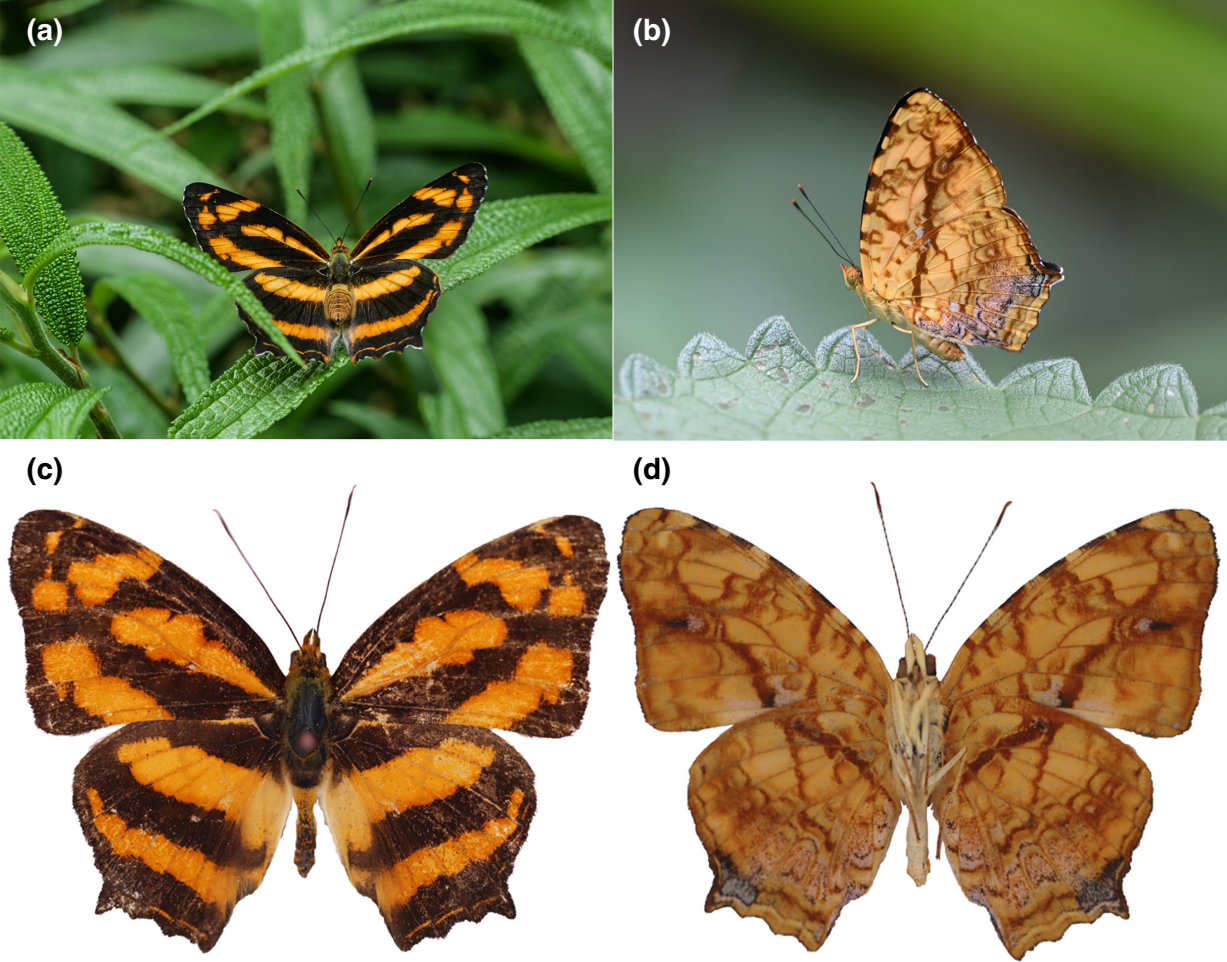
*Symbrenthia lilaea*. (a, b) Live adult, dorsal and lateral habitus; (c, d) Dorsal and ventral habitus. © Lan Zhang.

The Oriental nymphalid butterfly genus *Symbrenthia* Hübner, 1819 (Nymphalinae), commonly called jesters, belongs to the tribe Nymphalini which has 14 recognized species (Fric et al., 2004, 2022; Kunte, 2010), mainly distributed in the Oriental region and reaching New Guinea and the eastern Palaearctic (Bozano & Floriani, 2012). *Symbrenthia lilaea* Hewitson, 1864, the common jester, is distributed primarily in India, China, Nepal, Bhutan, Myanmar, and Pakistan (Mehra et al., 2018). An adult female of *S. lilaea* was collected from *Debregeasia* sp. (Urticaceae) on July 7, 2019 at Kuankuoshui National Nature Reserve (28°17′25″N, 107°12′09″E; collected by Zhanglan) in Zunyi, Guizhou Province, China (Figure 1). A single leg of *S. lilaea* was stored in 100% ethanol at 20°C and sent to Guangzhou Ruike Gene Technology Co. for mitogenome extraction and sequencing. For Illumina sequencing, genomic DNA was isolated using TIANamp Genomic DNA Kit (Tiangen). The Illumina sequencing library was generated using Truseq Nano DNA HT Sample Prep Kit (Illumina). The complete mitogenome was sequenced using high‐throughput sequencing on the Illumina Novaseq 6000 platform with an average insert size of 350 bp and a paired‐end 150 bp (PE 150) sequencing strategy to generate a sequencing data not less than 2 GB. Raw reads were trimmed of adapters using Trimmomatic (Bolger et al., 2014).

### 2.2 Sequence assembly, annotation, and analyses

**FIGURE 2:**
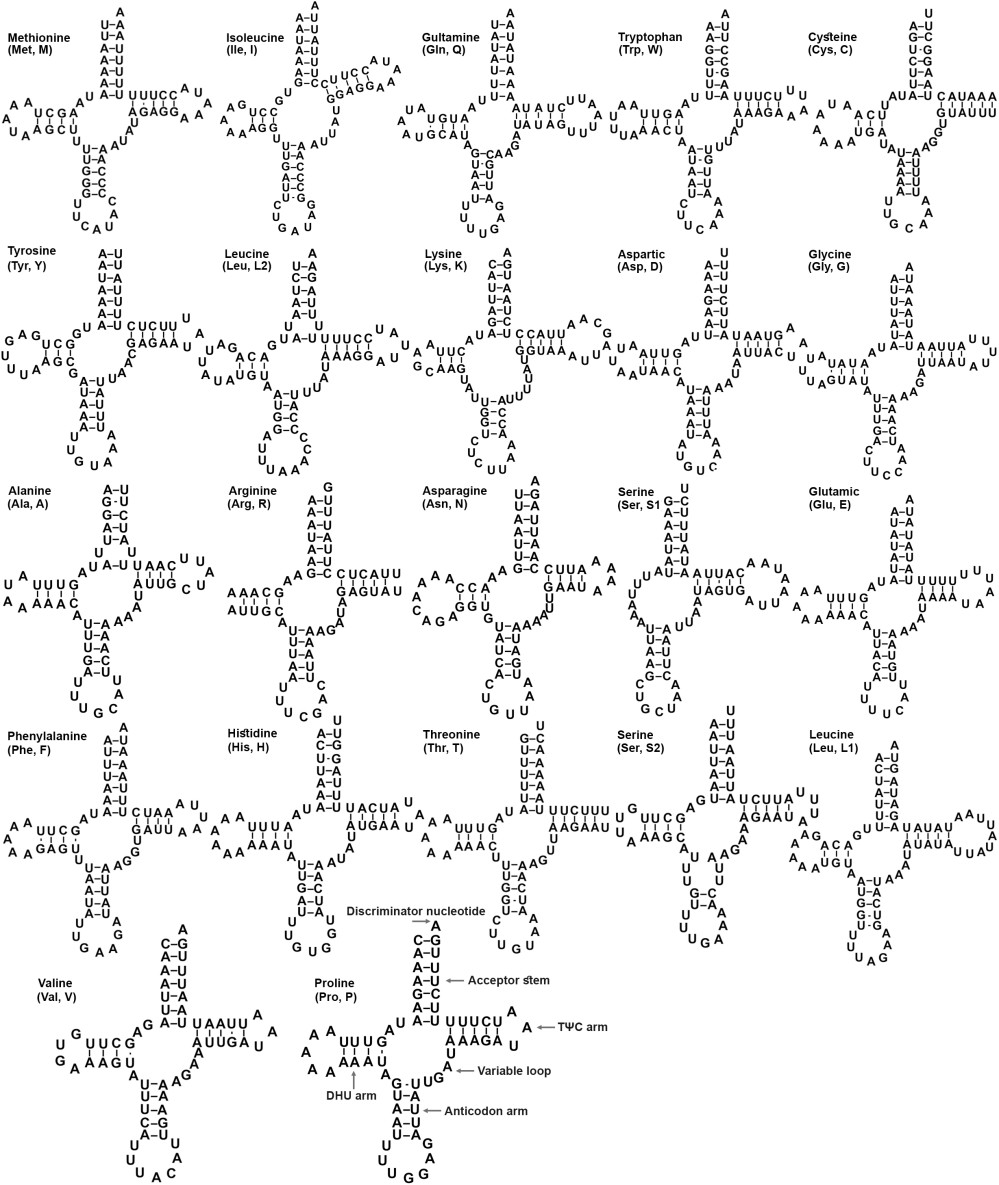
Predicted secondary cloverleaf structures for the tRNAs of the newly sequenced *Symbrenthia lilaea*.

Sequenced data for *S. lilaea* (OR161883) was aligned with the complete mitochondrial genome of *Melitaea cinxia* (HM243592) as a reference in GENEIOUS (v. 10.2.3) (Kearse et al., 2012). The 13 protein‐coding genes (PCGs) and two ribosomal RNA (rRNA) genes of *Symbrenthia lilaea* were confirmed by alignment with all published mitogenomes of Nymphalinae. The 22 transfer RNA (tRNA) genes were annotated using the MITOS web server (http://mitos.bioinf.unileipzig.de/index.py; Bernt et al., 2013). The secondary structure of each predicted tRNA obtained from the MITOS web server was manually plotted in Adobe Photoshop CS 6.0 (Figure 2). The graphical map of the circular genome and annotation were made using the CGView Server (http://stothard.afns.ualberta.ca/cgview_server/; Grant & Stothard, 2008). MEGA Version 7.0 was used to analyze the base composition of the complete mitogenome. The strand asymmetry was calculated by using the formulas: GC‐skew = [(G − C)/(G + C)] and AT‐skew = [(A − T)/(A + T)] (Perna & Kocher, 1995).

### 2.3 Phylogenetic analyses

In order to reconstruct the phylogenetic relationships among the nymphalid subfamilies and tribes of Nymphalinae, we retrieved the complete mitochondrial genome sequences of 80 species from 11 subfamilies available on NCBI, including the new sequence data of *S. lilaea* as ingroup, and six outgroup species from the closely related families Lycaenidae (three species), and Pieridae (three species). The GenBank accession numbers, mitogenome size, locality data, and references for the species studies are presented in Table 1. The complete mitogenome genes were extracted using PhyloSuite (v1.2.2; Zhang et al., 2020). The nucleotide sequences of 13 individual PCGs were aligned using MUSCLE (Edgar, 2004) in MEGA 7.0 (Kumar et al., 2016), and the stop codons were manually deleted. Alignments of 13 PCGs were concatenated using FASconCAT_v1.0 (Kück & Meusemann, 2010). The amino acid sequences of 13 PCGs were aligned using MUSCLE in MEGA 7.0. RNA gene alignment was conducted using MAFFT and then trimmed using trimAl to align the sequence. Finally, we concatenated the aligned sequenced of each gene to generate five datasets using PhyloSuite: (1) PCG123 (nucleotide data of 13 PCGs: 10,944 bp); (2) PCG123‐rRNA (nucleotide data of the 13 PCGs and rRNA genes: 12,708 bp); (3) PCG12 (nucleotide data of the 13 PCGs excluding the third codon position: 7296 bp); (4) PCG12‐RNA (nucleotide data of the 13 PCGs excluding the third codon position and RNA genes: 10,252 bp); (5) PCG_AA (amino acid sequences of the 13 PCGs: 3648 bp). The concatenated aligned sequences of each dataset were then converted into phylip and nexus formats using MESQUITE version 3.61 (Maddison & Maddison, 2019) for the Maximum likelihood (ML) and Bayesian inference (BI) analyses.

**TABLE 1:** List of mitogenomes used for the phylogenetic analyses in this study.

S. No		Family	Subfamily	Tribe	Species	Accession number	Bp	Locality	References
1	Ingroup	Nymphalidae	Apaturinae		*Apatura ilia*	JF437925	15,242	China (Zhejiang)	Chen et al. (2012)
2	Apaturinae		*Apatura metis*	NC015537	15,236	China (Heilongjiang)	Zhang et al. (2012)
3	Apaturinae		*Sasakia funebris*	AP011824	15,244	China (Sichuan)	Wang et al. (2013)
4	Apaturinae		*Sasakia charonda*	NC014224	15,244		GenBank
5	Apaturinae		*Euripus nyctelius*	KR020515	15,417	China	Xuan et al. (2016)
6	Apaturinae		*Herona marathus*	KT279805	15,487	China	Wang et al. (2016)
7	Apaturinae		*Timelaea maculata*	KC572131	15,178	China	Cao et al. (2013)
8	Biblidinae	Ageroniini	*Hamadryas epinome*	KM378244	15,207	France	Cally et al. (2016)
9	Charaxinae	Charaxini	*Polyura arja*	KF590540	15,363	China (Yunnan)	Wu et al. (2014)
10	Calinaginae		*Calinaga davidis*	HQ658143	15,267	China (Anhui)	Xia et al. (2011)
11	Danainae	Danaini	*Danaus plexippus*	KC836923	15,314	Mexico	Servín‐Garcidueñas and Martínez‐Romero (2014)
12	Danainae	Danaini	*Euploea mulciber*	HQ378507	15,166	China (Yunnan)	Hao et al. (2013)
13	Danainae	Danaini	*Euploea core*	KF590546	15,192	China	Wu et al. (2014)
14	Danainae	Danaini	*Euploea midamus*	KJ866207	15,187	China	GenBank
15	Danainae	Danaini	*Danaus chrysippus*	KF690637	15,236	China	Gan, Sun, et al. (2014)
16	Danainae	Danaini	*Ideopsis similis*	KJ476729	15,200	China (Anhui)	Gan, Chen, et al. (2014)
17	Danainae	Danaini	*Parantica sita*	KF590544	15,211	China (Taiwan)	Wu et al. (2014)
18	Danainae	Danaini	*Tirumala limniace*	KJ784473	15,285	China	Gan et al. (2016)
19	Heliconiinae	Acraeini	*Acraea issoria*	GQ376195	15,245	China (Anhui)	Hu et al. (2010)
20	Heliconiinae	Argynnini	*Argynnis childreni*	KF590547	15,131	China (Fujian)	Wu et al. (2014)
21	Heliconiinae	Argynnini	*Argynnis hyperbius*	JF439070	15,156	China (Anhui)	Wang et al. (2011)
22	Heliconiinae	Argynnini	*Fabriciana nerippe*	JF504707	15,140	Korea	Kim et al. (2011)
23	Heliconiinae	Argynnini	*Issoria lathonia*	HM243590	15,172	China	GenBank
24	Heliconiinae	Heliconiini	*Heliconius pachinus*	NC024741	15,369	China	Huang, Dai, et al. (2014)
25	Heliconiinae	Heliconiini	*Heliconius hecale*	KM068091	15,338	China	Shen and Wang (2016)
26	Heliconiinae	Heliconiini	*Heliconius cydno*	KM208636	15,367		Qian (2016)
27	Heliconiinae	Heliconiini	*Heliconius melpomene rosina*	KP100653	15,327		Meng et al. (2016)
28	Libytheinae		*Libythea lepita*	HQ378508	15,164	China (Anhui)	Hao et al. (2013)
29	Limenitidinae	Adoliadini	*Euthalia irrubescens*	KF590527	15,366	China (Taiwan)	Wu et al. (2014)
30	Limenitidinae	Adoliadini	*Tanaecia julii*	KF590548	15,316	China (Yunnan)	Wu et al. (2014)
31	Limenitidinae	Adoliadini	*Lexias dirtea*	KF590531	15,250	Malaysia (Sarawak)	Wu et al. (2014)
32	Limenitidinae	Adoliadini	*Dophla evelina*	KF590532	15,320	Malaysia (Sarawak)	Wu et al. (2014)
33	Limenitidinae	Adoliadini	*Abrota ganga*	KF590536	15,356	China (Guanxi)	Wu et al. (2014)
34	Limenitidinae	Limenitidini	*Athyma opalina*	KF590551	15,240	China (Taiwan)	Wu et al. (2014)
35	Limenitidinae	Limenitidini	*Athyma kasa*	KF590524	15,230	Philippines (Luzon)	Wu et al. (2014)
36	Limenitidinae	Limenitidini	*Athyma selenophora*	KF590525	15,200	China (Hainan)	Wu et al. (2014)
37	Limenitidinae	Limenitidini	*Athyma selenophora*	KF590529	15,208	China (Taiwan)	Wu et al. (2014)
38	Limenitidinae	Limenitidini	*Athyma cama*	KF590526	15,269	China (Taiwan)	Wu et al. (2014)
39	Limenitidinae	Limenitidini	*Athyma asura*	KF590542	15,181	China (Taiwan)	Wu et al. (2014)
40	Limenitidinae	Limenitidini	*Athyma perius*	KF590528	15,277	China (Taiwan)	Wu et al. (2014)
41	Limenitidinae	Limenitidini	*Athyma sulpitia*	JQ347260	15,268	China (Jiangxi)	Tian et al. (2012)
42	Limenitidinae	Limenitidini	*Pandita sinope*	KF590530	15,257	Malaysia (Sarawak)	Wu et al. (2014)
43	Limenitidinae	Limenitidini	*Sumalia daraxa*	KF590549	15,167	Thailand (Phitsanulok)	Wu et al. (2014)
44	Limenitidinae	Limenitidini	*Parasarpa dudu*	KF590537	15,236	China (Taiwan)	Wu et al. (2014)
45	Limenitidinae	Perthenini	*Parthenos sylvia*	KF590550	15,249	China (Yunnan)	Wu et al. (2014)
46	Limenitidinae	Neptini	*Bhagadatta austenia*	KF590545	15,615	China (Guandong)	Wu et al. (2014)
47	Limenitidinae	Neptini	*Neptis soma*	KF590533	15,130	China (Taiwan)	Wu et al. (2014)
48	Limenitidinae	Neptini	*Neptis philyra*	KF590552	15,164	China (Taiwan)	Wu et al. (2014)
49	Limenitidinae	Neptini	*Pantoporia hordonia*	KF590534	15,603	China (Hong Kong)	Wu et al. (2014)
50	Nymphalinae	Junoniini	*Yoma sabina*	KF590535	15,330	China (Taiwan)	Wu et al. (2014)
51	Nymphalinae	Junoniini	*Junonia almana*	KF590539	15,256	Japan (Okinawa)	Wu et al. (2014)
52	Nymphalinae	Junoniini	*Junonia iphita*	KU577290	15,190		GenBank
53	Nymphalinae	Junoniini	*Junonia lemonias*	KP941756	15,230	China: Yunnan	McCullagh and Marcus (2015)
54	Nymphalinae	Junoniini	*Junonia orithya*	KF199862	15,214	China	Shi, Huang, et al. (2013)
55	Nymphalinae	Junoniini	*Junonia villida*	KX267582	15,210	Australia	GenBank
56	Nymphalinae	Kallimini	*Catacroptera cloanthe*	MW722786	15,204	Tanzania	Lalonde (2022)
57	Nymphalinae	Kallimini	*Kallima inachus*	JN857943	15,183	China (Guangxi)	Qin et al. (2012)
58	Nymphalinae	Kallimini	*Kallima paralekta*	MW192438	15,200	Thailand (Pattaya)	Aguila et al. (2021)
59	Nymphalinae	Kallimini	*Mallika jacksoni*	MT704828	15,193	Uganda	GenBank
60	Nymphalinae	Melitaeini	*Mellicta ambigua*	MK252271	15,205	Korea	Kim et al. (2021)
61	Nymphalinae	Melitaeini	*Melitaea cinxia‐1*	GQ398377	15,170	China	GenBank
62	Nymphalinae	Melitaeini	*Melitaea cinxia‐1*	HM243592	15,162	China	GenBank
63	Nymphalinae	Nymphalini	Symbrenthia lilaea	**OR161883**	15,726	China	**Present study**
64	Nymphalinae	Nymphalini	*Nymphalis ladakensis*	MN732892	15,222	China: Qinhai	Chen et al. (2020)
65	Nymphalinae	Nymphalini	*Nymphalis io*	KM592970	15,250		GenBank
66	Nymphalinae	Nymphalini	*Nymphalis c‐aureum*	MF407452	15,209	China: Nanjing	Shi et al. (2018)
67	Nymphalinae	Nymphalini	*Vanessa indica*	MG736927	15,191	China: Anhui	Lu et al. (2018)
68	Nymphalinae	Nymphalini	*Araschnia levana*	MT712075	15,207	Belgium	Alexiuk et al. (2020)
69	Nymphalinae	Nymphalini	*Smyrna blomfildia*	MZ151338	15,149	Peru	Alexiuk et al. (2021)
70	Nymphalinae	Victorini	*Anartia jatrophae saturata*	MT712074	15,297	Dominican Republic	Payment et al. (2020)
71	Pseudergolinae	Pseudergolini	*Dichorragia nesimachus*	KF590541	15,355	China (Taiwan)	Wu et al. (2014)
72	Satyrinae	Melanitini	*Melanitis phedima*	KF590538	15,142	China (Taiwan)	Wu et al. (2014)
73	Satyrinae	Melanitini	*Melanitis leda*	JF905446	15,122	China	Shi, Zhao, et al. (2013)
74	Satyrinae	Satyrini	*Neope pulaha*	KF590543	15,209	China (Taiwan)	Wu et al. (2014)
75	Satyrinae	Satyrini	*Lasiommata deidamia*	MG880214	15,244	China (Anhui)	Sun et al. (2021)
76	Satyrinae	Satyrini	*Ypthima akragas*	KF590553	15,227	China (Taiwan)	Wu et al. (2014)
77	Satyrinae	Satyrini	*Melanargia asiatica*	KF906486	15,142		Huang, Hao, et al. (2014)
78	Satyrinae	Satyrini	*Pararge aegeria*	KJ547676	15,240		Teixeira da Costa (2016)
79	Satyrinae	Satyrini	*Triphysa phryne*	KF906487	15,143		Zhang et al. (2016)
80	Satyrinae	Satyrini	*Hipparchia autonoe*	GQ868707	15,489	South Korea (Island Jeju)	Kim et al. (2010)
81	Outgroup	Lycaenidae	Theclinae	Theclini	*Coreana raphaelis*	DQ102703	15,314	Korea (Gwangwon)	Kim et al. (2006)
82			Theclinae	Theclini	*Protantigius superans*	HQ184265	15,248	Korea (Gangwon‐do)	Kim et al. (2011)
83			Aphnaeinae	Aphnaeini	*Cigaritis takanonis*	HQ184266	15,349	Korea (Gangwon‐do)	Kim et al. (2011)
84		Pieridae	Pierinae	Pierini	*Pieris melete*	EU597124	15,140	China (Jiangxi)	Hong et al. (2009)
85			Pierinae	Pierini	*Pieris rapae*	HM156697	15,157	China (Auhui)	Mao et al. (2010)
86			Pierinae	Pierini	*Aporia crataegi*	JN796473	15,140	Mongolia	Mao et al. (2010)

Phylogenetic trees were reconstructed using the ML and BI methods. ML analyses were performed in IQ‐TREE (Nguyen et al., 2015) using ultrafast bootstrap with 5000 replicates as implemented on the webserver (http://iqtree.cibiv.univie.ac.at/), and the BI analyses were performed in MrBayes v.3.2.6 (Ronquist et al., 2012) implemented in PhyloSuite software with various data partition schemes and best‐fitting models determined by PartitionFinder (Lanfear et al., 2017; Appendix S2, Tables S1–S10). The BI analyses contains four simultaneous Markov chain Monte Carlo (MCMC) runs of 2 million generations, and sampled every 1000 generations. The initial 25% of the sampled data were discarded as burn‐in. Other parameters were kept at default settings. The phylogenetic trees were visualized and edited in the Interactive Tree of Life (iTOL: https://itol.embl.de) version 5 (Letunic & Bork, 2021).

## 3 RESULTS

### 3.1 Genome structure and nucleotide composition

**FIGURE 3:**
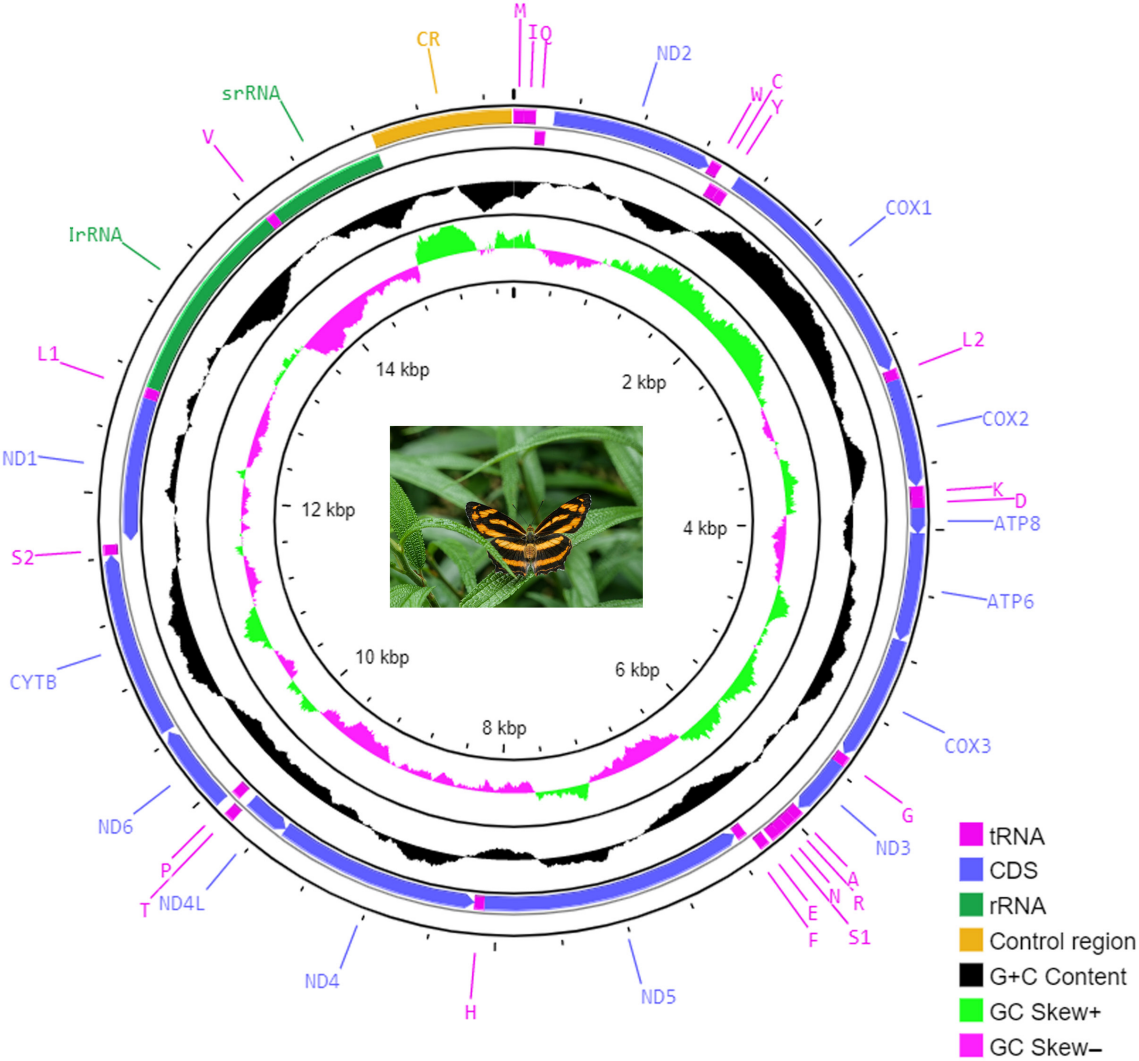
The circular mitogenome map of *Symbrenthia lilaea*.

The complete mitochondrial genome of *S. lilaea* is sequenced, annotated, and analyzed in this study. The complete mitochondrial genome of *S. lilaea* is 15,726 bp in length, containing 37 genes (22 transfer RNA genes, two ribosomal RNA genes, and 13 protein‐encoding genes) and the control region (Table 2), with the same ancestral gene order and arrangement in Nymphalidae. The circular mitogenome map of *S. lilaea* is shown in Figure 3. The overall nucleotide composition in *S. lilaea* is 87.7% versus 12.3% of A + T content and G + C content, respectively (A = 35.7%, T = 52.0%, C = 6.4%, G = 5.9%). The A + T content of isolated PCGs, tRNAs, rRNAs, and control region were all above 77% (Table 3).

**TABLE 2:** Organization of the complete mt genome in *Symbrenthia lilaea*.

Gene	Direction	Location	Size (bp)	IGN*	Anticodon	Start codon	Stop codon
tRNA^Met^	F	1―67	67	0	CAT		
tRNA^Ile^	F	71―135	65	3	GAT		
tRNA^Gln^	R	137―205	69	1	TTG		
ND2	F	266―1258	993	60		ATT	TTT
tRNA^Trp^	F	1266―1332	67	−2	TCA		
tRNA^Cys^	R	1325―1390	66	−8	GCA		
tRNA^Tyr^	R	1391―1456	66	0	GTA		
COI	F	1465―2988	1524	8		TGA	AAC
tRNA^Leu(UUR)^	F	2989―3055	67	0	TAA		
COII	F	3057―3728	672	1		ATG	AAT
tRNA^Lys^	F	3732―3801	70	3	CTT		
tRNA^Asp^	F	3801―3865	67	−1	GTC		
ATP8	F	3867―4025	159	1		ATT	ATA
ATP6	F	4031―4690	660	5		ATG	TAT
COIII	F	4720―5493	774	29		CAT	AAT
tRNA^Gly^	F	5498―5564	67	4	TCC		
ND3	F	5566―5916	350	1		ATC	AAT
tRNA^Ala^	F	5917―5979	63	0	TGC		
tRNA^Arg^	F	5980―6041	62	0	TCG		
tRNA^Asn^	F	6042―6107	66	0	GTT		
tRNA^Ser(AGN)^	F	6106―6166	61	−2	GCT		
tRNA^Glu^	F	6191―6256	66	24	TTC		
tRNA^Phe^	R	6271―6335	65	14	GAA		
ND5	R	6341―8053	1712	5		TAA	AAT
tRNA^His^	R	8068―8133	66	14	GTG		
ND4	R	8148―9473	1326	14		TAT	CAT
ND4L	R	9478―9750	273	4		AAT	TAT
tRNA^Thr^	F	9768―9831	64	17	TGT		
tRNA^Pro^	R	9832―9896	65	0	TGG		
ND6	F	9917―10429	513	20		ATC	CAT
CytB	F	10436―11575	1140	6		ATA	TTT
tRNA^Ser(UCN)^	F	11583―11647	65	7	TGA		
ND1	R	11673―12587	915	25		TAA	TAT
tRNA^Leu(CUN)^	R	12604―12673	70	16	TAG		
lrRNA	R	12653―14022	1370	−21			
tRNA^Val^	R	14018―14081	64	−5	TAC		
srRNA	R	14082―14854	773	0			
Control region	–	14855―15726	872	0			

**TABLE 3:** Base composition of *Symbrenthia lilaea*.

	Size (bp)	T%	C%	A%	G%	A + T%	G + C%	AT skew	GC skew
Genome	15,726	41.9	11.9	38.7	7.5	80.6	19.4	−0.039	−0.227
PCGs	10,971	45.3	10.6	33.5	10.6	78.8	21.2	−0.041	0
rRNA	2114	39.7	5	45	10.3	84.7	15.3	0.063	0.346
tRNAs	1445	39.5	8.7	41.8	10	81.3	18.7	0.028	0.069
Control region	875	52	6.4	35.7	5.9	87.7	12.3	−0.186	0.041

### 3.2 Phylogenetic analyses

**FIGURE 4:**
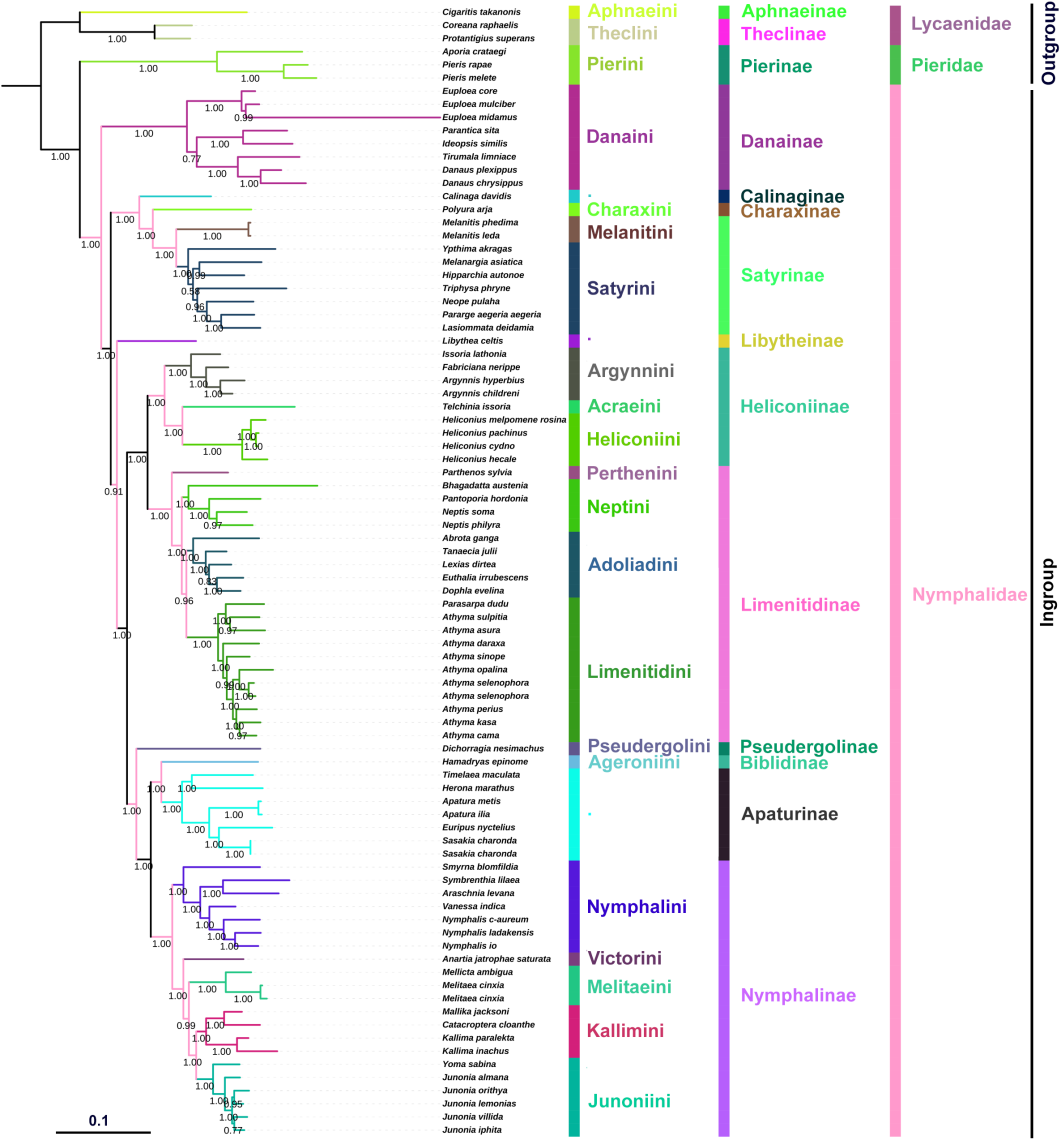
The phylogenetic relationships of Nymphalidae using the Bayesian inference (BI) analysis method based on the concatenated nucleotide sequences of PCG123 + 2 rRNAs datasets. Numbers on each node correspond to the posterior probability (PP) values.

**FIGURE 5:**
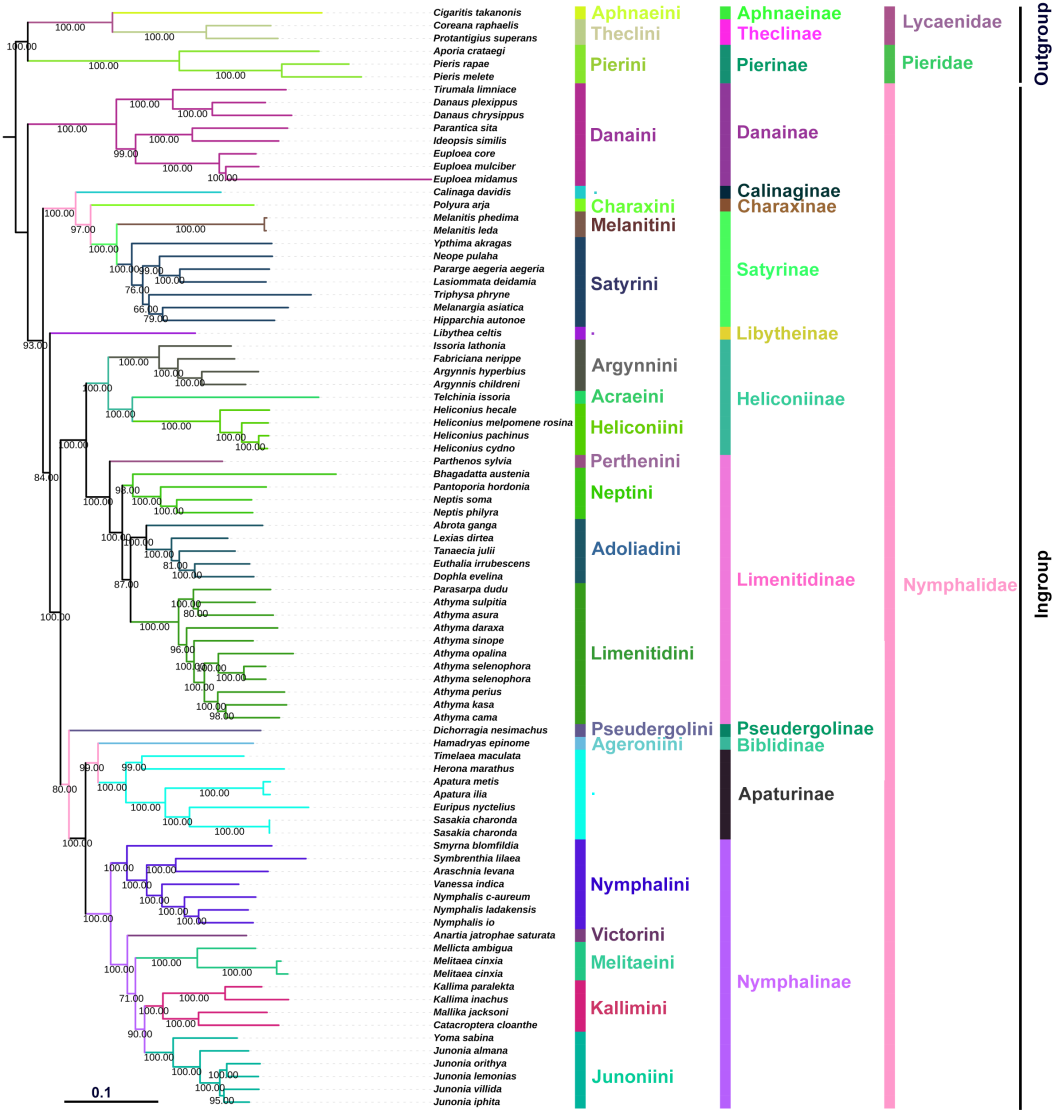
The phylogenetic relationships of Nymphalidae using the maximum likelihood (ML) analysis method based on the concatenated nucleotide sequences of PCG12 + 2 rRNAs + 22 tRNAs datasets. Numbers on each node correspond to the bootstrap values.

We discussed the basal clade position in Nymphalidae and tribal‐level relationships among the subfamily Nymphalinae based on 80 ingroups (Nymphalinae 21 species; Limenitidinae 21 spp.; Heliconiinae 9 spp.; Satyrinae 9 spp.; Danainae 8 spp.; Apaturinae 7 species; Biblidinae 1 sp.; Calinaginae 1 sp.; Charaxinae 1 sp.; Pseudergolinae 1 sp.; Libytheinae 1 sp.), and six outgroup species (Lycaenidae 3 spp.; Pieridae 3 spp.) available on NCBI, including the new sequence data of *S. lilaea*. The phylogenetic relationships were reconstructed by using five different datasets of the complete mitochondrial genome through the ML and BI methods. Ten phylogenetic trees were obtained based on BI and ML analyses, but here we selected the best tree among these methods with identical topologies with high supporting values for Danainae as the basal clade and sister to the remaining nymphalid subfamilies. Phylogenetic relationships among the nine subfamilies in three clades are recovered as monophyletic with high support values in both BI and ML analyses, which are generally consistent with previously published studies (Figures 4 and 5; Wu et al., 2014). These clades include the satyrine clade (Calinaginae, Charaxinae, and Satyrinae), the heliconiine clade (Heliconiinae and Limenitidinae), and the nymphaline clade (Nymphalinae, Biblidinae, Apaturinae, Pseudergolinae, and Cyrestinae). However, Libytheinae, the morphologically unique subfamily, was either recovered as a sister to Danainae followed by the satyrine clade or sister to the nymphaline + heliconiine clades.

## 4 DISCUSSION

The basal clade of Nymphalidae has always been controversial and still confounds systematic and evolutionary biologists, with either Danainae or Libytheinae as a basal clade position based on evidence from morphological, molecular, host plants, and biogeographical studies (Espeland et al., 2018; Freitas & Brown, 2004; Shi et al., 2015). In the cladistic analyses, host plants, one mitochondrial (COI), and one or two nuclear genes (EF‐1α and wingless), Libytheinae has always been proposed as a basal clade to all Nymphalidae based on features from the immature stage (lacks horns or spines) and adult female characters (foreleg with tarsal claws; Brower, 2000; Freitas & Brown, 2004; Wahlberg et al., 2003). However, Shi et al. (2015) proposed Danainae as the basal group to all nymphalid subfamilies based on adult wing character (connected medius one vein and radius vein) and Libytheinae as the sister to the nymphaline + heliconiine clade based on the slightly closed or open discal cell of the forewing. This hypothesis is supported in various molecular phylogenetic studies based on both nuclear and mitochondrial genomes, which supported the danaine clade as the basal group and the uncertain phylogenetic relationship of Libytheinae among the subfamilies (Chazot et al., 2021; Hao et al., 2013; Shi et al., 2015; Wu et al., 2014).

However, different phylogenetic hypotheses for the position of Libytheinae among the clades of Nymphalidae have been proposed based on different mitogenome datasets and analyses methods: (1) 13 PCGs: a sister group to the satyrine clade [NJ, 65] or the nymphaline + heliconiine clades [BI, 0.97] (Hao et al., 2013); (2) 13 PCGs + 2 rRNAs + 22 tRNAs, 13PCGs + 2rRNAs, 13 PCGs: a sister to the nymphaline + heliconiine clades (ML, 55; BI, 0.74); (3) 13 PCGs + 2 rRNAs: a sister to the nymphaline + heliconiine clades (ML, 33–77; BI, 1.00–0.81; Shi et al., 2015). Herein, we recovered Danainae as the basal clade of all nymphalid subfamilies with high support values of posterior probability in BI (0.91) and bootstrap in ML (84) analyses, and reconfirmed the paraphyletic Libytheinae, which is not stable among the other nymphalid subfamilies (Figures 4 and 5). Libytheinae is either recovered as a sister to the danaine clade followed by the satyrine clade (posterior probability = 0.61; ML bootstrap = 53–65) or sister to the nymphaline + heliconiine clades (posterior probability = 0.91; ML bootstrap = 40–84) in various analyses by using the following five different datasets: (1) PCG123 + 2 rRNAs + 22 tRNAs; (2) PCG123 + 2 rRNAs; (3) 13 PCGs; (4) PCG12 + 2 rRNAs +22 tRNAs; (5) PCG123_AA (Appendix S1, Figures S1–S8). This subfamily‐level relationship is consistent with previous published mitogenomic studies by Hao et al. (2013), Wu et al. (2014), and Shi et al. (2015), and supported the danaine clade as the basal group to all nymphalid subfamilies with high support values in all analyses. However, the phylogenetic position of Libytheinae remains unresolved. So far, only one mitogenome of Libytheinae is available on NCBI (*Libythea lepita*: Accession number, HQ378508); in order to better resolve the phylogenetic position of Libytheinae among the nymphalid subfamilies, new mitochondrial genomes must be updated, and various datasets with different analyses should be reinvestigated in future studies.

The monophyletic Nymphalinae has been recovered in several morphological and molecular studies (Kim et al., 2021; Shi et al., 2015; Su et al., 2017; Wahlberg et al., 2003, 2005; Wahlberg & Wheat, 2008; Wu et al., 2014); however, the internal relationships among its tribes are largely dependent on the use of different molecular datasets, analyses methods, and taxon sampling. In addition to large‐scale studies on both morphological and molecular datasets on the subfamily Nymphalinae, we reinvestigate tribal‐level relationships by incorporating new *Symbrenthia* mitogenome sequence data with different molecular datasets for the first time. Based on the concatenated mitogenome sequence data of 21 species from five tribes, the current study supports the monophyly of Nymphalinae and resolves tribal‐level relationships with high support values in both BI and ML analyses. However, the phylogenetic relationships within Nymphalinae are significantly affected by taxon sampling among different tribes (Su et al., 2017; Wu et al., 2014). We supported the monophyletic Nymphalini as a sister clade to Victorini, Melitaeini, and Kallimini + Junoniini with high support in BI and ML analyses using five different datasets (Appendix S1, Figures S1–S8), which is consistent with previously published morphological and molecular studies (Wahlberg et al., 2005). However, our present analyses do not support for the Kallimini to be a sister to Junoniini + Melitaeini (Wu et al., 2014), or sister to the clade comprising Melitaeini and Junoniini (Shi et al., 2015).

## 5 CONCLUSIONS

The phylogenetic relationships among the nymphalid subfamilies and tribes of Nymphalinae were reconstructed by using five different datasets of the complete mitochondrial genome through the ML and BI methods. Here, we included 80 ingroup species in 11 subfamilies that recovered consistent topologies and supported Danainae as a basal clade to all subfamilies. Libytheinae is either recovered as a sister to the danaine clade followed by the satyrine clade or sister to the nymphaline + heliconiine clades in various analyses by using five different datasets: (1) PCG123 + 2 rRNAs + 22 tRNAs; (2) PCG123 + 2 rRNAs; (3) 13 PCGs; (4) PCG12 + 2 rRNAs + 22 tRNAs; (5) PCG123_AA. The subfamily Nymphalinae also appeared as monophyletic and Nymphalini as a basal clade and is sister to Vic‐torini, Melitaeini, and Kallimini + Junoniini.

## AUTHOR CONTRIBUTIONS

**Muhammad Asghar Hassan:** Conceptualization (equal); data curation (equal); methodology (equal); resources (equal); software (equal); writing – original draft (equal); writing – review and editing (equal). **Rongrong Shen:** Conceptualization (equal); data curation (equal); investigation (equal); methodology (equal); software (equal); visualization (equal); writing – original draft (equal). **Lan Zhang:** Conceptualization (equal); data curation (equal); formal analysis (equal); methodology (equal); resources (equal); visualization (equal); writing – original draft (equal). **Taslima Sheikh:** Data curation (equal); methodology (equal); writing – original draft (equal); writing – review and editing (equal). **Jichun Xing:** Conceptualization (equal); investigation (equal); resources (equal); supervision (equal); visualization (equal); writing – original draft (equal); writing – review and editing (equal).

## FUNDING INFORMATION

This work was supported by the Ministry of Science and Technology of the People's Republic of China (Grant no. 2005DKA21402), Biodiversity Survey and Assessment Project of the Ministry of Ecology and Environment, China (Grant no. 2019HJ2096001006).

## CONFLICT OF INTEREST STATEMENT

The authors declare no conflict of interest.

## Supporting information


Appendix S1.

Appendix S2.

## Data Availability

The mitogenome sequences of *Symbrenthia lilaea* has been deposited on GenBank and assigned accession number (OR161883) is provided.

## References

[ece310263-bib-0001] Aguila, C. P. , Aikens, R. M. , Ateliey, P. K. , Buhr, H. M. , Castro, M. G. , Chua, R. J. , Dayal, N. , Deane, H. N. , Dennehy, B. , Esenbekova, M. , Fay, J. L. , Gair, C. , Gordon, B. R. , Huh, S. , Ishrar, F. , Jonson, E. B. , Kaur, C. F. , Kokolo, C. , Lanyon, K. , … Marcus, J. M. (2021). The complete mitochondrial genome of the Indian leafwing butterfly *Kallima paralekta* (Insecta: Lepidoptera: Nymphalidae). Mitochondrial DNA Part B, 6, 274–277. 33553643 10.1080/23802359.2020.1862000 PMC7850329

[ece310263-bib-0002] Alexiuk, M. R. , Lalonde, M. M. L. , & Marcus, J. M. (2021). Phylogenetic analysis of the complete mitochondrial genome of the Blomfild's beauty butterfly *Smyrna blomfildia* (Fabricius 1781) (Insecta: Lepidoptera: Nymphalidae: Nymphalini). Mitochondrial DNA Part B, 6, 3199–3201. 34660902 10.1080/23802359.2021.1989337 PMC8519521

[ece310263-bib-0003] Alexiuk, M. R. , Marcus, J. M. , & Lalonde, M. M. L. (2020). The complete mitochondrial genome and phylogenetic analysis of the European map butterfly *Araschnia levana* (Insecta: Lepidoptera: Nymphalidae). Mitochondrial DNA Part B, 5, 3246–3248. 33458126 10.1080/23802359.2020.1810163 PMC7782646

[ece310263-bib-0004] Bernt, M. , Donath, A. , Jühling, F. , Externbrink, F. , Florentz, C. , Fritzsch, G. , Pütz, J. , Middendorf, M. , & Stadler, P. F. (2013). MITOS: Improved de novo metazoan mitochondrial genome annotation. Molecular Biology and Evolution, 69(2), 313–319. 10.1016/j.ympev.2012.08.023 22982435

[ece310263-bib-0005] Bolger, A. M. , Lohse, M. , & Usadel, B. (2014). Trimmomatic: A flexible trimmer for Illumina sequence data. Bioinformatics, 30, 2114–2120. 24695404 10.1093/bioinformatics/btu170 PMC4103590

[ece310263-bib-0006] Bozano, G. C. , & Floriani, A. (2012). Guide to the butterflies of the palaearctic region. In T. Nymphalini & J. Kallimini (Eds.), Nymphalidae part V. subfamily Nymphalinae (p. 90). Omnes Artes.

[ece310263-bib-0007] Brower, A. V. (2000). Phylogenetic relationships among the Nymphalidae (Lepidoptera) inferred from partial sequences of the wingless gene. Proceedings of the Royal Society of London. Series B: Biological Sciences, 267, 1201–1211. 10.1098/rspb.2000.1129 PMC1690664 10902686

[ece310263-bib-0008] Cally, S. , Lhuillier, E. , Iribar, A. , Garzón‐Orduña, I. , Coissac, E. , & Murienne, J. (2016). Shotgun assembly of the complete mitochondrial genome of the neotropical cracker butterfly *Hamadryas epinome* . Mitochondrial DNA Part A, 27, 1864–1866. 10.3109/19401736.2014.971262 25319307

[ece310263-bib-0009] Cao, T. W. , Wang, J. P. , Xuan, S. B. , Zhang, M. , Guo, Y. P. , & Ma, E. B. (2013). Analysis of complete mitochondrial genome of *Timelaea maculate* (Lepidoptera, Nymphalidae). Acta Zootaxonomica Sinica, 38, 468–475.

[ece310263-bib-0010] Chazot, N. , Condamine, F. , Dudas, G. , Peña, C. , Kodandaramaiah, U. , Matos‐Maraví, P. , Aduse‐Poku, K. , Elias, M. , Warren, A. D. , Lohman, D. , Penz, C. , DeVries, P. , Fric, Z. F. , Nylin, S. , Müller, C. , Kawahara, A. Y. , Silva‐Brandao, K. , Lamas, G. , Kleckova, I. , … Wahlberg, N. (2021). Conserved ancestral tropical niche but different continental histories explain the latitudinal diversity gradient in brushfooted butterflies. Nature Communications, 12, 5717. 10.1038/s41467-021-25906-8 PMC8481491 34588433

[ece310263-bib-0011] Chen, K. , Si, C. , Pan, Z. , & Hao, J. (2020). The complete mitochondrial genome of *Aglais ladakensis* (Lepidoptera: Nymphalidae: Nymphalinae). Mitochondrial DNA Part B, 5, 639–641. 33366682 10.1080/23802359.2019.1711224 PMC7748854

[ece310263-bib-0102] Chen, M. , Tian, L. L. , Shi, Q. H. , Cao, T. W. , & Hao, J. S. (2012). Complete mitogenome of the Lesser Purple Emperor *Apatura ilia* (Lepidoptera: Nymphalidae: Apaturinae) and comparison with other nymphalid butterflies. Zoological Research, 33(2), 191–201. 22467396 10.3724/SP.J.1141.2012.02191

[ece310263-bib-0012] Edgar, R. C. (2004). MUSCLE: Multiple sequence alignment with high accuracy and high throughput. BMC Bioinformatics, 5, 113. 15318951 10.1186/1471-2105-5-113 PMC517706

[ece310263-bib-0013] Ehrlich, P. R. (1958). The comparative morphology, phylogeny and higher classification of the butterflies (Lepidoptera: Papilionoidea). The University of Kansas Science Bulletin, 39, 305–370.

[ece310263-bib-0014] Espeland, M. , Breinholt, J. , Willmott, K. R. , Warren, A. D. , Vila, R. , Toussaint, E. F. , Maunsell, S. C. , Aduse‐Poku, K. , Talavera, G. , Eastwood, R. , Jarzyna, M. A. , Guralnick, R. , Lohman, D. J. , Pierce, N. E. , & Kawahara, A. Y. (2018). A comprehensive and dated phylogenomic analysis of butterflies. Current Biology, 28, 770–778. 29456146 10.1016/j.cub.2018.01.061

[ece310263-bib-0015] Freitas, A. V. L. , & Brown, K. S. J. (2004). Phylogeny of the nymphalidae (Lepidoptera). Systematic Biology, 53, 363–383. 15503668 10.1080/10635150490445670

[ece310263-bib-0016] Fric, Z. F. , Konvicka, M. , & Zrzavy, J. (2004). Red & black or black & white?: Phylogeny of the Araschnia butterflies (Lepidoptera: Nymphalidae) and evolution of seasonal polyphenism. Journal of Evolutionary Biology, 17, 265–278. 15009260 10.1111/j.1420-9101.2003.00681.x

[ece310263-bib-0017] Fric, Z. F. , Martinkova, B. , Rindos, M. , Bartonova, A. S. , Wahlberg, N. , & Maresova, J. P. (2022). Molecular phylogeny and biogeography of the genus *Symbrenthia* (Lepidoptera, Nymphalidae) correlates with the past geography of the oriental region. Molecular Phylogenetics and Evolution, 177, 107605. 35952836 10.1016/j.ympev.2022.107605

[ece310263-bib-0018] Gan, S. S. , Chen, Y. H. , Zuo, N. , Xia, C. C. , & Hao, J. S. (2016). The complete mitochondrial genome of *Tirumala limniace* (Lepidoptera: Nymphalidae: Danainae). Mitochondrial DNA, 27, 1096–1098. 24975850 10.3109/19401736.2014.930839

[ece310263-bib-0019] Gan, S. S. , Chen, Y. H. , Zuo, N. , Zhang, W. , & Hao, J. S. (2014). The complete mitochondrial genome of *Ideopsis similis* (Lepidoptera: Nymphalidae: Danainae). Mitochondrial DNA, 27, 531–532. 24708111 10.3109/19401736.2014.905842

[ece310263-bib-0020] Gan, S. S. , Sun, X. Y. , Gai, Y. H. , & Hao, J. S. (2014). The complete mitochondrial genome of *Danaus chrysippus* (Lepidoptera: Nymphalidae: Danainae). Mitochondrial DNA, 26, 819–820. 24409860 10.3109/19401736.2013.855909

[ece310263-bib-0021] Grant, J. R. , & Stothard, P. (2008). The CGView server: A comparative genomics tool for circular genomes. Nucleic Acids Research, 36(Suppl_2), W181–W184. 18411202 10.1093/nar/gkn179 PMC2447734

[ece310263-bib-0022] Hao, J. S. , Sun, M. E. , Shi, Q. H. , Sun, X. Y. , Shao, L. L. , & Yang, Q. (2013). Complete mitogenomes of *Euploea mulciber* (Nymphalidae: Danainae) and *Libythea celtis* (Nymphalidae: Libytheinae) and their phylogenetic implications. ISRN Genome, 2013, 1–14.

[ece310263-bib-0023] Hong, G. , Jiang, S. , Yu, M. , Yang, Y. , Li, F. , Xue, F. , & Wei, Z. (2009). The complete nucleotide sequence of the mitochondrial genome of the cabbage butterfly, *Artogeia melete* (Lepidoptera: Pieridae). Acta Biochimica et Biophysica Sinica, 41, 446–455. 19499147 10.1093/abbs/gmp030

[ece310263-bib-0024] Hu, J. , Zhang, D. , Hao, J. , Huang, D. , Cameron, S. , & Zhu, C. (2010). The complete mitochondrial genome of the yellow coaster, *Acraea issoria* (Lepidoptera: Nymphalidae: Heliconiinae: Acraeini): Sequence, gene organization and a unique tRNA translocation event. Molecular Biology Reports, 37, 3431–3438. 20091125 10.1007/s11033-009-9934-3

[ece310263-bib-0025] Huang, D. Y. , Hao, J. S. , Zhang, W. , Su, T. J. , Wang, Y. , & Xu, X. F. (2014). The complete mitochondrial genome of *Melanargia asiatica* (Lepidoptera: Nymphalidae: Satyrinae). Mitochondrial DNA Part A, 27, 806–808. 10.3109/19401736.2014.919452 24866032

[ece310263-bib-0026] Huang, Z. H. , Dai, P. F. , & Zhao, G. F. (2014). The complete mitochondrial genome of *Heliconius pachinus* (Insecta: Lepidoptera: Nymphalidae). Mitochondrial DNA Part A, 27, 1251–1252. 10.3109/19401736.2014.945542 25090391

[ece310263-bib-0027] Kawahara, A. Y. (2009). Phylogeny of snout butterflies (Lepidoptera: Nymphalidae: Libytheinae): Combining evidence from the morphology of extant, fossil, and recently extinct taxa. Cladistics, 25, 263–278. 34879612 10.1111/j.1096-0031.2009.00251.x

[ece310263-bib-0028] Kearse, M. , Moir, R. , Wilson, A. , Stones‐Havas, S. , Cheung, M. , Sturrock, S. , Buxton, S. , Cooper, A. , Markowitz, S. , Duran, C. , Thierer, T. , Ashton, B. , Meintjes, P. , & Drummond, A. (2012). Geneious basic: An integrated and extendable desktop software platform for the organization and analysis of sequence data. Bioinformatics, 28, 1647–1649. 22543367 10.1093/bioinformatics/bts199 PMC3371832

[ece310263-bib-0029] Kim, I. , Lee, E. M. , Seol, K. Y. , Yun, E. Y. , Lee, Y. B. , Hwang, J. S. , & Jin, B. R. (2006). The mitochondrial genome of the Korean hairstreak, *Coreana raphaelis* (Lepidoptera: Lycaenidae). Insect Molecular Biology, 15, 217–225. 16640732 10.1111/j.1365-2583.2006.00630.x

[ece310263-bib-0030] Kim, M. J. , Chua, M. , Parka, J. S. , Kim, S. S. , & Kima, I. (2021). Complete mitochondrial genome of the summer heath fritillary butterfly, *Mellicta ambigua* (Lepidoptera: Nymphalidae). Mitochondrial DNA Part B, 6, 1603–1605. 34027067 10.1080/23802359.2021.1917318 PMC8118395

[ece310263-bib-0031] Kim, M. J. , Jeong, H. C. , Kim, S. R. , & Kim, I. (2011). Complete mitochondrial genome of the nerippe fritillary butterfly, *Argynnis nerippe* (Lepidoptera: Nymphalidae). Mitochondrial DNA, 22, 86–88. 22040072 10.3109/19401736.2011.624604

[ece310263-bib-0032] Kim, M. J. , Wan, X. , Kim, K. G. , Hwang, J. S. , & Kim, I. (2010). Complete nucleotide sequence and organization of the mitogenome of endangered *Eumenis autonoe* (Lepidoptera: Nymphalidae). African Journal of Biotechnology, 9, 735–754.

[ece310263-bib-0033] Kück, P. , & Meusemann, K. (2010). FASconCAT: Convenient handling of data matrices. Molecular Phylogenetics and Evolution, 56, 1115–1118. 20416383 10.1016/j.ympev.2010.04.024

[ece310263-bib-0034] Kumar, S. , Stecher, G. , & Tamura, K. (2016). MEGA7: Molecular evolutionary genetics analysis version 7.0 for bigger datasets. Molecular Biology and Evolution, 33, 1870–1874. 27004904 10.1093/molbev/msw054 PMC8210823

[ece310263-bib-0101] Kunte, K. (2010). Rediscovery of the federally protected Scarce Jester Butterfly *Symbrenthia silana* de Nicéville, 1885 (Nymphalidae: Nymphalinae) from the Eastern Himalaya and Garo Hills, northeastern India. Journal of Threatened Taxa, 2(5), 858–866.

[ece310263-bib-0035] Lalonde, M. M. L. (2022). The complete mitochondrial genome of the pirate butterfly *Catacroptera cloanthe* (Stoll, 1781) (Insecta: Lepidoptera: Nymphalidae: Kallimini). Mitochondrial DNA Part B, 7, 306–308. 35111943 10.1080/23802359.2022.2030818 PMC8803096

[ece310263-bib-0036] Lanfear, R. , Frandsen, P. B. , Wright, A. M. , Senfeld, T. , & Calcott, B. (2017). PartitionFinder 2: New methods for selecting partitioned models of evolution for molecular and morphological phylogenetic analyses. Molecular Biology and Evolution, 34, 772–773. 28013191 10.1093/molbev/msw260

[ece310263-bib-0037] Letunic, I. , & Bork, P. (2021). Interactive tree of life (iTOL) v5: An online tool for phylogenetic tree display and annotation. Nucleic Acids Research, 49(W1), W293–W296. 33885785 10.1093/nar/gkab301 PMC8265157

[ece310263-bib-0038] Lu, Y. , Liu, N. , Xu, L. , Fang, J. , & Wang, S. (2018). The complete mitochondrial genome of *Vanessa indica* and phylogenetic analyses of the family Nymphalidae. Genes & Genomics, 40, 1011–1022. 29949077 10.1007/s13258-018-0709-x

[ece310263-bib-0039] Maddison, W. P. , & Maddison, D. R. (2019). Mesquite: A modular system for evolutionary analysis . Version 3.61. http://www.mesquiteproject.org

[ece310263-bib-0040] Mao, Z. H. , Hao, J. S. , Zhu, G. P. , Hu, J. , Si, M. M. , & Zhu, C. D. (2010). Sequencing and analysis of the complete mitochondrial genome of *Pieris rapae* Linnaeus (Lepidoptera: Pieridae). Acta Entomologica Sinica, 53, 1295–1304.

[ece310263-bib-0041] McCullagh, B. S. , & Marcus, J. M. (2015). The complete mitochondrional genome of lemon pansy, *Junonia lemonias* (Lepidoptera: Nymphalidae: Nymphalinae). Journal of Asia‐Pacific Entomology, 18, 749–755.

[ece310263-bib-0042] Mehra, D. , Kirti, J. S. , & Sidhu, A. K. (2018). Taxonomic review of the tribe Nymphalini (Lepidoptera: Nymphalidae: Nymphalinae) from western Himalaya, India with special emphasis on external genitalic attributes. Entomon, 43, 237–256.

[ece310263-bib-0043] Meng, Z. , Lei, C. , Chen, X. , & Jiang, S. (2016). Complete mitochondrial genome sequence of *Heliconius melpomene rosina* (Insecta: Lepidoptera: Nymphalidae). Mitochondrial DNA, 27, 3911–3912. 25484166 10.3109/19401736.2014.987261

[ece310263-bib-0044] Nguyen, L. T. , Schmidt, H. A. , von Haeseler, A. , & Minh, B. Q. (2015). IQ‐TREE: A fast and effective stochastic algorithm for estimating maximum‐likelihood phylogenies. Molecular Biology and Evolution, 32, 268–274. 25371430 10.1093/molbev/msu300 PMC4271533

[ece310263-bib-0045] Nylin, S. , Nyblom, K. , Ronquist, F. , Janz, N. , Belicek, J. , & Kallersjo, M. (2001). Phylogeny of Polygonia, Nymphalis and related butterflies (Lepidoptera: Nymphalidae): A total‐evidence analysis. Zoological Journal of the Linnean Society, 132, 441–468.

[ece310263-bib-0047] Payment, J. E. , Marcus, J. M. , & Lalonde, M. M. L. (2020). Phylogenetic analysis of the complete mitochondrial genome of the white peacock butterfly *Anartia jatrophae saturata* (Insecta: Lepidoptera: Nymphalidae). Mitochondrial DNA Part B, 5, 3690–3692. 33367069 10.1080/23802359.2020.1832929 PMC7655040

[ece310263-bib-0048] Perna, N. T. , & Kocher, T. D. (1995). Patterns of nucleotide composition at fourfold degenerate sites of animal mitochondrial genomes. Journal of Molecular Evolution, 41, 353–358. 7563121 10.1007/BF00186547

[ece310263-bib-0049] Qian, Z. Q. (2016). The complete mitogenome of the cydno longwing *Heliconius cydno* (Insecta: Lepidoptera: Nymphalidae). Mitochondrial DNA Part A, 27, 1453–1454. 10.3109/19401736.2014.953089 25162223

[ece310263-bib-0050] Qin, X. M. , Guan, Q. X. , Zeng, D. L. , Qin, F. , & Li, H. M. (2012). Complete mitochondrial genome of *Kallima inachus* (Lepidoptera: Nymphalidae: Nymphalinae): Comparison of *K. inachus* and *Argynnis hyperbius* . Mitochondrial DNA, 23, 318–320. 22708853 10.3109/19401736.2012.684093

[ece310263-bib-0051] Ronquist, F. , Teslenko, M. , van der Mark, P. , Ayres, D. L. , Darling, A. , Höhna, S. , Larget, B. , Liu, L. , Suchard, M. A. , & Huelsenbeck, J. P. (2012). MrBayes 3.2: Efficient Bayesian phylogenetic inference and model selection across a large model space. Systematic Biology, 61, 539–542. 22357727 10.1093/sysbio/sys029 PMC3329765

[ece310263-bib-0105] Servín‐Garcidueñas, L. E. , & Martínez‐Romero, E. (2014). Complete mitochondrial genome recovered from the gut metagenome of overwintering monarch butterflies, *Danaus plexippus* (L.) Lepidoptera: Nymphalidae, Danainae. Mitochondrial DNA, 25(6), 427–428. 23834084 10.3109/19401736.2013.809441

[ece310263-bib-0052] Shen, Q. Q. , & Wang, L. (2016). The complete mitochondrial genome sequence of *Heliconius hecale* (Insecta: Lepidoptera: Nymphalidae). Mitochondrial DNA Part A, 27, 1291–1292. 10.3109/19401736.2014.945561 25090389

[ece310263-bib-0053] Shi, Q. H. , Huang, D. Y. , Wang, Y. L. , & Hao, J. S. (2013). The complete mitochondrial genome of blue pansy, *Junonia orithya* (Lepidoptera: Nymphalidae: Nymphalinae). Mitochondrial DNA, 26, 245–246. 24021009 10.3109/19401736.2013.823182

[ece310263-bib-0054] Shi, Q. H. , Sun, X. Y. , Wang, Y. L. , Hao, J. S. , & Yang, Q. (2015). Morphological characters are compatible with mitogenomic data in resolving the phylogeny of nymphalid butterflies (Lepidoptera: Papilionoidea: Nymphalidae). PLoS ONE, 10, e0124349. 25860387 10.1371/journal.pone.0124349 PMC4393276

[ece310263-bib-0055] Shi, Q. H. , Xing, J. H. , Liu, X. H. , & Hao, J. S. (2018). The complete mitochondrial genome of comma, *Polygonia c‐aureum* (Lepidoptera: Nymphalidae: Nymphalinae). Mitochondrial DNA Part B, 3, 53–55. 10.1080/23802359.2017.1419091 PMC7799567 33474063

[ece310263-bib-0056] Shi, Q. H. , Zhao, F. , Hao, J. S. , & Yang, Q. (2013). Complete mitochondrial genome of the common evening brown, *Melanitis leda* Linnaeus (Lepidoptera: Nymphalidae: Satyrinae). Mitochondrial DNA, 24, 492–494. 23464597 10.3109/19401736.2013.770501

[ece310263-bib-0057] Su, C. Y. , Shi, Q. H. , Sun, X. Y. , Ma, J. Y. , Li, C. X. , Hao, J. S. , & Yang, Q. (2017). Dated phylogeny and dispersal history of the butterfly subfamily Nymphalinae (Lepidoptera: Nymphalidae). Scientific Reports, 7, 8799. 28821757 10.1038/s41598-017-08993-w PMC5562872

[ece310263-bib-0058] Sun, Y. X. , Chen, C. , Geng, X. X. , & Li, J. (2021). Complete mitochondrial genome of *Lasiommata deidamia* and its phylogenetic implication to subfamily Satyrinae (Lepidoptera: Nymphalidae). Mitochondrial DNA Part B, 6, 2943–2945. 34553051 10.1080/23802359.2021.1955029 PMC8451643

[ece310263-bib-0059] Teixeira da Costa, L. F. (2016). The complete mitochondrial genome of *Parage aegeria* (Insecta: Lepidoptera: Papilionidae). Mitochondrial DNA Part A, 27, 551–552. 10.3109/19401736.2014.905853 24708119

[ece310263-bib-0060] Tian, L. L. , Sun, X. Y. , Chen, M. , Gai, Y. H. , Hao, J. S. , & Yang, Q. (2012). Complete mitochondrial genome of the five‐dot sergeant *Parathyma sulpitia* (Nymphalidae: Limenitidinae) and its phylogenetic implications. Zoological Research, 33, 133–143. 22467387 10.3724/SP.J.1141.2012.02133

[ece310263-bib-0061] Wahlberg, N. , Brower, A. V. , & Nylin, S. (2005). Phylogenetic relationships and historical biogeography of tribes and genera in the subfamily Nymphalinae (Lepidoptera: Nymphalidae). Biological Journal of the Linnean Society, 86, 227–251.

[ece310263-bib-0062] Wahlberg, N. , Leneveu, J. , Kodandaramaiah, U. , Peña, C. , Nylin, S. , Freitas, A. V. , & Brower, A. V. (2009). Nymphalid butterflies diversify following near demise at the cretaceous/tertiary boundary. Proceedings of the Royal Society B: Biological Sciences, 276, 4295–4302. 10.1098/rspb.2009.1303 PMC2817107 19793750

[ece310263-bib-0063] Wahlberg, N. , Weingartner, E. , & Nylin, S. (2003). Towards a better understanding of the higher systematics of Nymphalidae (Lepidoptera: Papilionoidea). Molecular Phylogenetics and Evolution, 28, 473–484. 12927132 10.1016/s1055-7903(03)00052-6

[ece310263-bib-0064] Wahlberg, N. , & Wheat, C. W. (2008). Genomic outposts serve the phylogenomic pioneers: Designing novel nuclear markers for genomic DNA extractions of Lepidoptera. Systematic Biology, 57, 231–242. 18398768 10.1080/10635150802033006

[ece310263-bib-0104] Wang, J. P. , Cao, T. W. , Xuan, S. B. , Wang, H. , Zhang, M. , & Ma, E. B. (2013). The complete mitochondrial genome of *Sasakia funebris* (Leech) (Lepidoptera: Nymphalidae) and comparison with other Apaturinae insects. Gene, 526(2), 336–343. 10.1016/j.gene.2013.05.036 23742889

[ece310263-bib-0103] Wang, J. P. , Xuan, S. B. , Cao, L. M. , Hao, J. S. , & Cao, T. W. (2016). The complete mitochondrial genome of the butterfly *Herona marathus* (Lepidoptera: Nymphalidae). Mitochondrial DNA Part A, 27(6), 4399–4400. 10.3109/19401736.2015.1089541 26477895

[ece310263-bib-0065] Wang, X. C. , Sun, X. Y. , Sun, Q. Q. , Zhang, D. X. , Hu, J. , Yang, Q. , & Hao, J. S. (2011). Complete mitochondrial genome of the laced fritillary *Argyreus hyperbius* (Lepidoptera: Nymphalidae). Zoological Research, 32, 465–475. 22006797 10.3724/SP.J.1141.2011.05465

[ece310263-bib-0066] Wu, L. W. , Lin, L. H. , Lees, D. C. , & Hsu, Y. F. (2014). Mitogenomic sequences effectively recover relationships within brush‐footed butterflies (Lepidoptera: Nymphalidae). BMC Genomics, 15, 1–17. 24923777 10.1186/1471-2164-15-468 PMC4070565

[ece310263-bib-0067] Xia, J. , Hu, J. , Zhu, G. P. , Zhu, C. D. , & Hao, J. S. (2011). Sequencing and analysis of the complete mitochondrial genome of *Calinaga davidis* Oberthür (Lepidoptera: Nymphalidae). Acta Entomologica Sinica, 54, 555–565.

[ece310263-bib-0068] Xuan, S. , Song, F. , Cao, L. , Wang, J. , Li, H. , & Cao, T. (2016). The complete mitochondrial genome of the butterfly *Euripus nyctelius* (Lepidoptera: Nymphalidae). Mitochondrial DNA Part A, 27, 2563–2565. 10.3109/19401736.2015.1038805 26024135

[ece310263-bib-0069] Zhang, D. , Gao, F. L. , Jakovlić, I. , Zou, H. , Zhang, J. , Li, W. X. , & Wang, G. T. (2020). PhyloSuite: An integrated and scalable desktop platform for streamlined molecular sequence data management and evolutionary phylogenetics studies. Molecular Ecology Resources, 20, 348–355. 31599058 10.1111/1755-0998.13096

[ece310263-bib-0106] Zhang, M. , Nie, X. P. , Cao, T. W. , Wang, J. , Li, T. , Zhang, X. N. , Guo, Y. P. , Ma, E. , & Zhong, Y. (2012). The complete mitochondrial genome of the butterfly *Apatura metis* (Lepidoptera: Nymphalidae). Molecular Biology Reports, 39, 6529–6536. 22311013 10.1007/s11033-012-1481-7

[ece310263-bib-0070] Zhang, W. , Gan, S. S. , Zuo, N. , Chen, C. H. , Wang, Y. , & Hao, J. S. (2016). The complete mitochondrial genome of *Triphysa phryne* (Lepidoptera: Nymphalidae: Satyrinae). Mitochondrial DNA Part A, 27(1), 474–475. 10.3109/19401736.2014.900673 24708130

