# Supplementary material for: Mitogenomic phylogeny of nymphalid subfamilies confirms the basal clade position of Danainae (Insecta: Lepidoptera: Nymphalidae)
Source: Ecol Evol. 2023 Jul 14;13(7):e10263. doi: 10.1002/ece3.10263 (PMC10346370; doi:10.1002/ece3.10263)
Supplement: Supplementary file 1 — Appendix S1. [file ECE3-13-e10263-s001.docx]

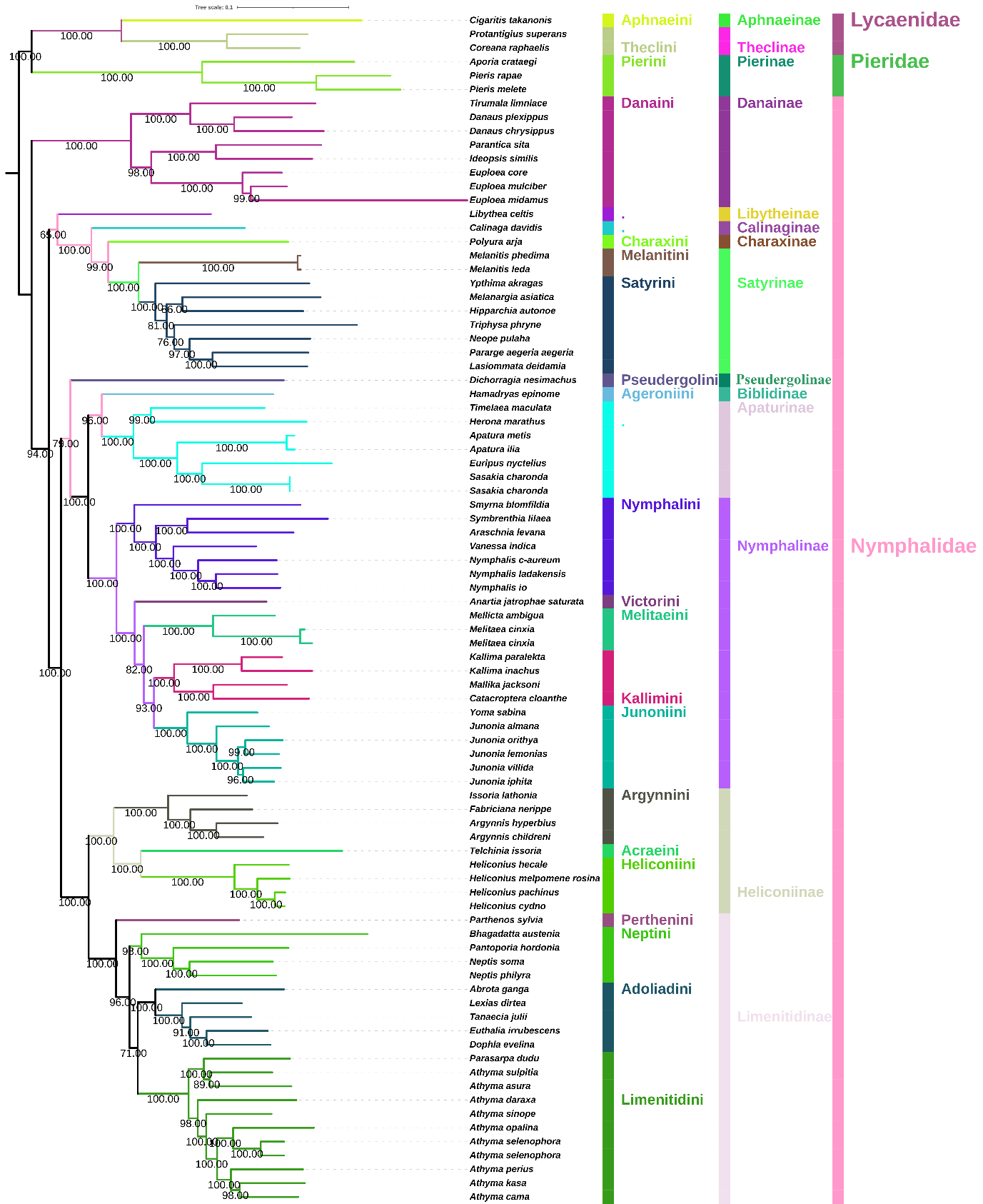


**Figure A1.** The phylogenetic tree produced by maximum likelihood analyses based on PCG123 + 2 rRNAs + 22 tRNAs datasets. Numbers at nodes are bootstrap support values (BS).


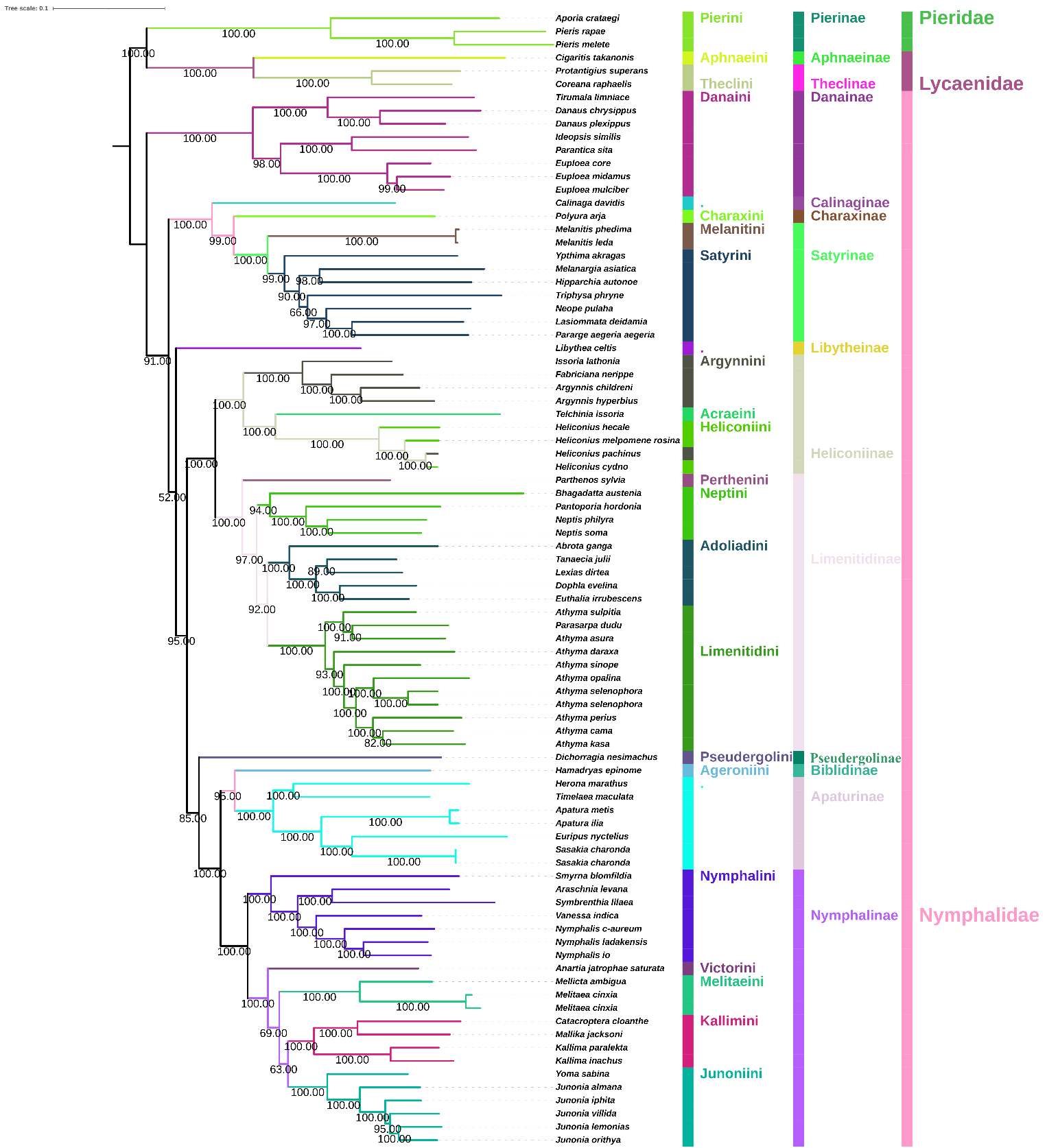


**Figure A2.** Phylogenetic tree produced by maximum likelihood analyses based on PCG123 dataset. Numbers at nodes are bootstrap support values (BS).


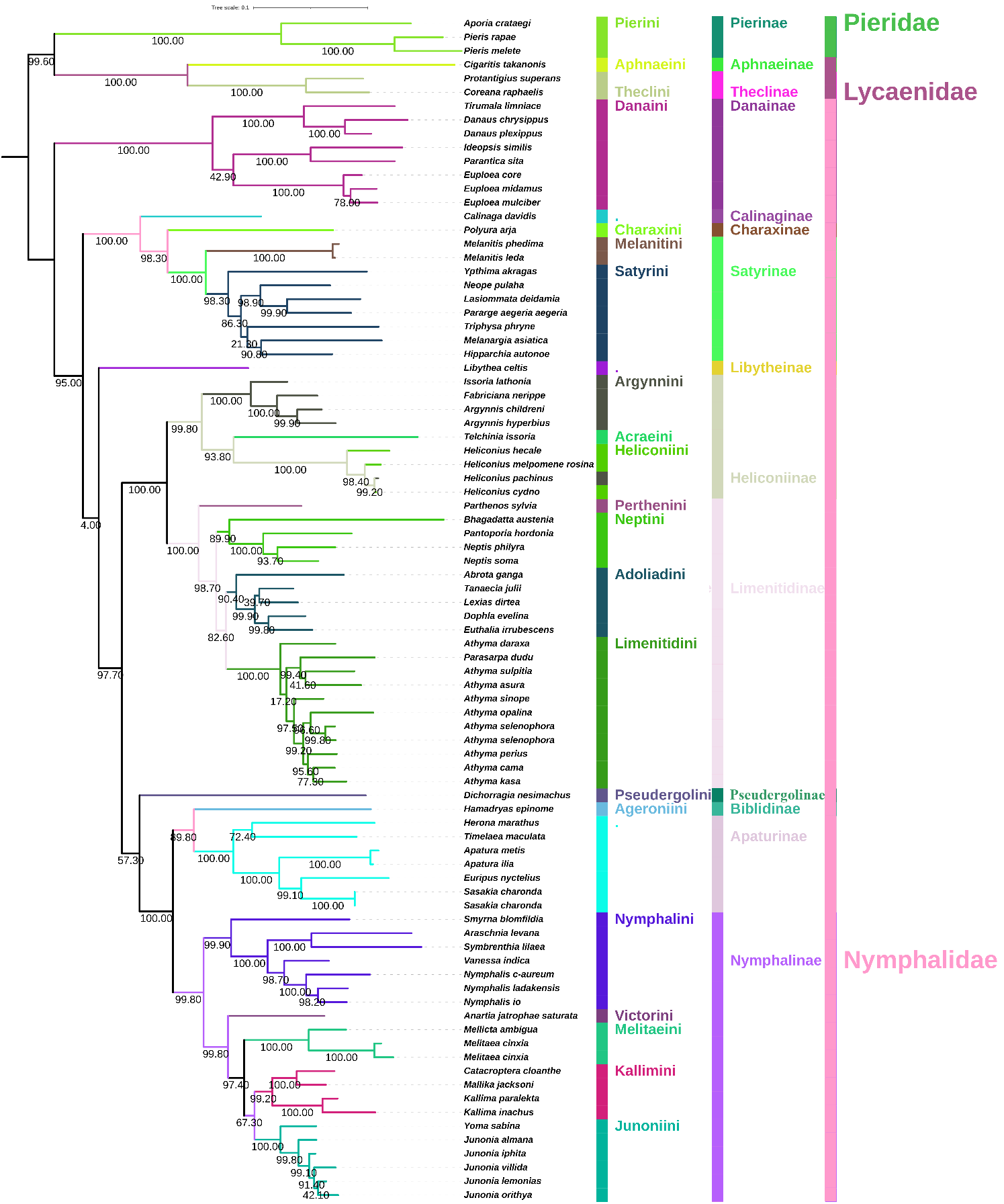


**Figure A3.** Phylogenetic tree produced by maximum likelihood analyses based on PCG123_AA dataset. Numbers at nodes are bootstrap support values (BS).


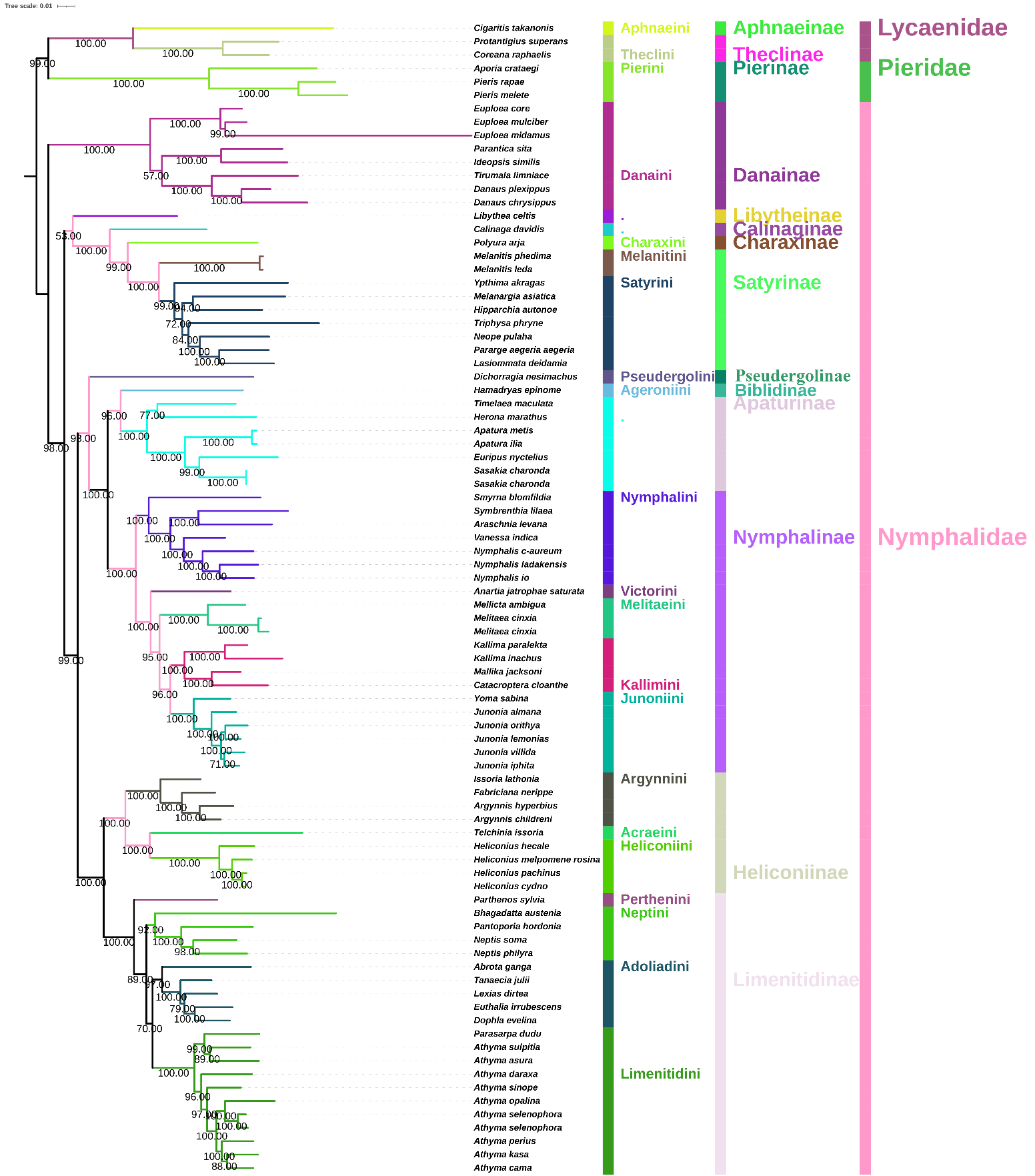


**Figure A4.** The phylogenetic tree produced by maximum likelihood analyses based on PCG12 + 2 rRNAs + 22 tRNAs datasets. Numbers at nodes are bootstrap support values (BS).


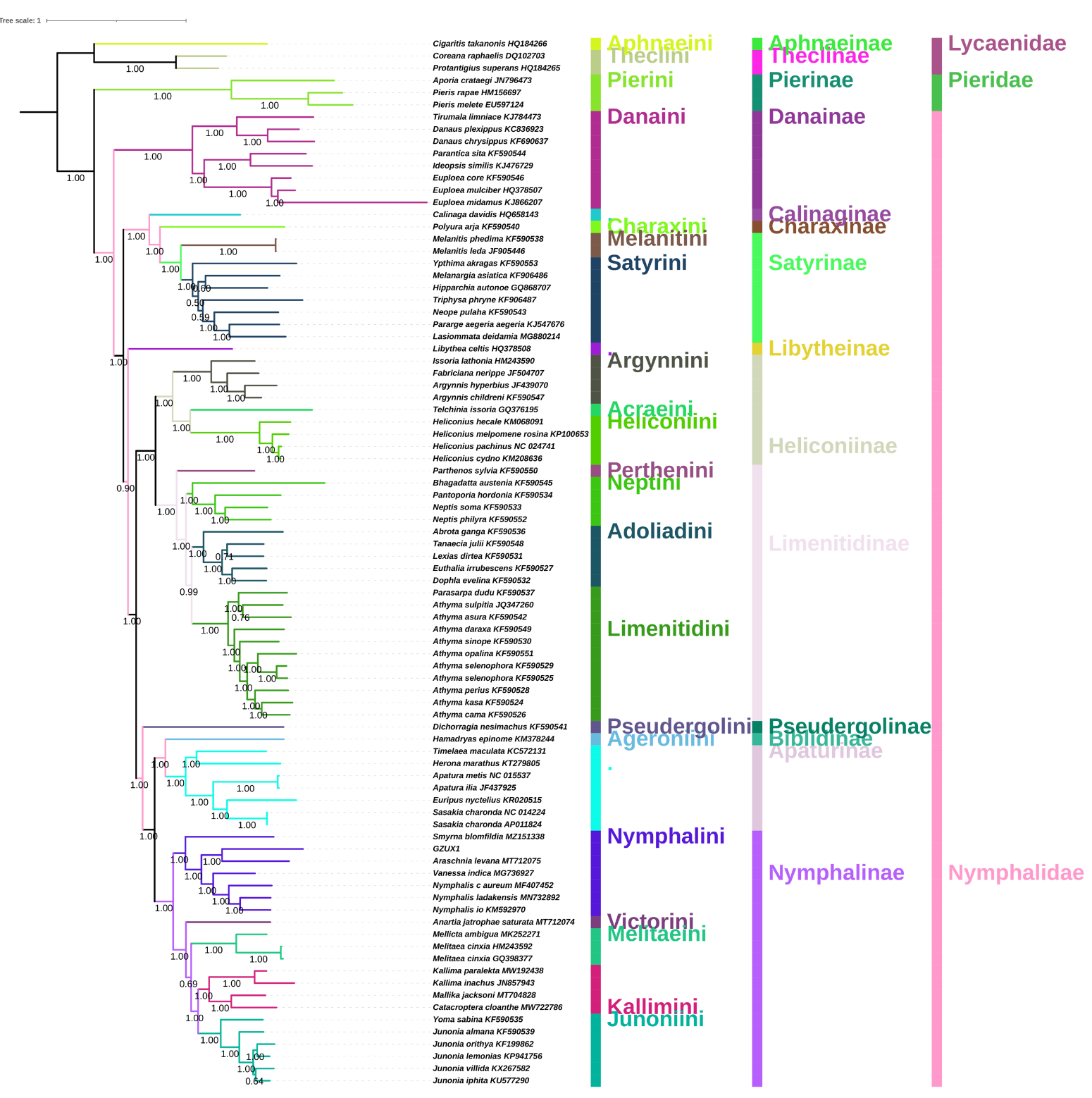


**Figure A5.** Phylogenetic tree produced by Bayesian inference analysis of the PCG123 + 2 rRNAs + 22 tRNAs datasets. Numbers at nodes are Bayesian posterior probability (BPP) support values.


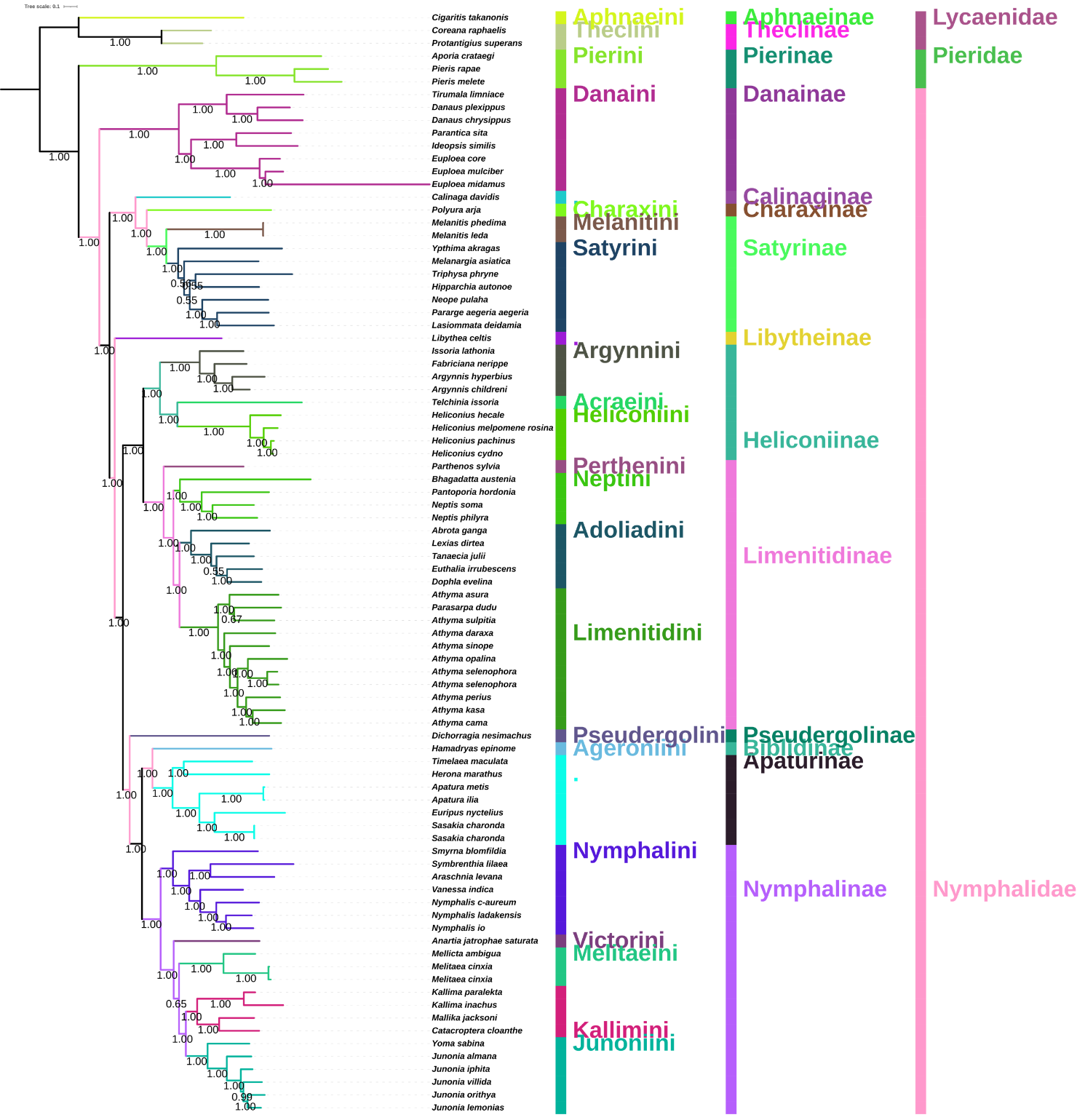


**Figure A6.** The phylogenetic tree produced by Bayesian inference analysis of the PCG123 + 2 rRNAs datasets. Numbers at nodes are Bayesian posterior probability (BPP) support values.


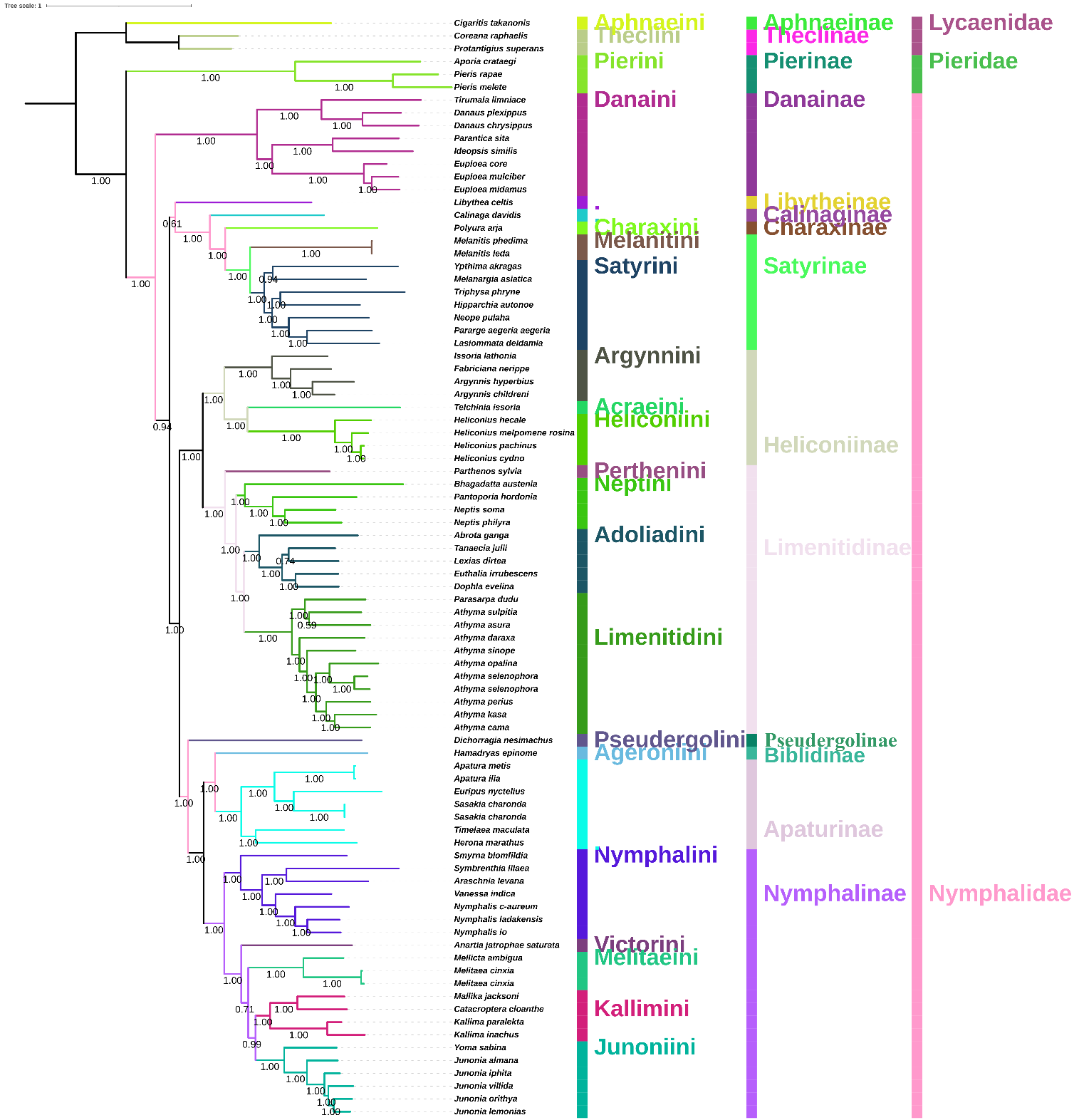


**Figure A7.** Phylogenetic tree produced by Bayesian inference analysis of the PCG123 dataset. Numbers at nodes are Bayesian posterior probability (BPP) support values.


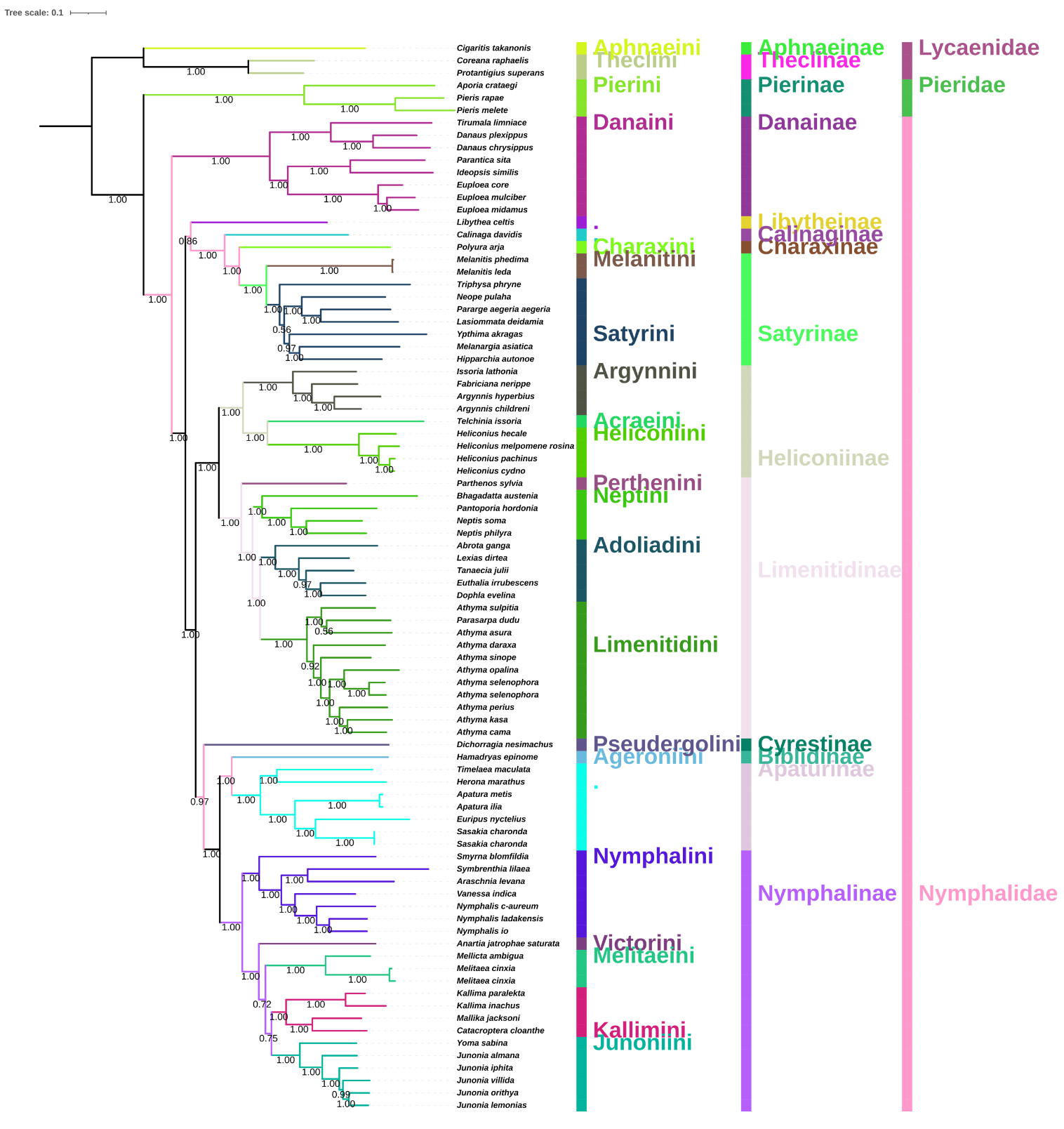
 **Figure A8.** The phylogenetic tree produced by Bayesian inference analysis of the PCG123_AA dataset. Numbers at nodes are Bayesian posterior probability (BPP) support values.
