# Supplementary material for: Mitogenomic phylogeny of nymphalid subfamilies confirms the basal clade position of Danainae (Insecta: Lepidoptera: Nymphalidae)
Source: Ecol Evol. 2023 Jul 14;13(7):e10263. doi: 10.1002/ece3.10263 (PMC10346370; doi:10.1002/ece3.10263)
Supplement: Supplementary file 2 — Appendix S2. [file ECE3-13-e10263-s002.docx]

**Table A1.** The best partitioning schemes and models for the Bayesian inference (BI) method based on PCG123 dataset selected by PartitionFinder.

| **Dataset** | Partitions | Partitioning scheme | Alignment | **Best model** | **No. of sites** |
| --- | --- | --- | --- | --- | --- |
| **PCG123** | P1 | atp6, cytb, cox3 | 7288-7956, 1-1149, 10267-11040 | GTR+I+G | 864 |
|  | P2 | cox1_pos2, cox2_pos2, atp6_pos2, cytb_pos2, cox3_pos2 | 8099-9612, 9614-10266, 7289-7956, 2-1149, 10268-11040 | GTR+I+G | 1587 |
|  | P3 | cox1_pos3, cytb_pos3 | 8100-9612, 3-1149 | GTR+I+G | 888 |
|  | P4 | nad4, nad4l, nad1, nad5 | 3712-5049, 3439-3711, 1150-2079, 5050-6765 | GTR+I+G | 1419 |
|  | P5 | nad4l_pos2, nad5_pos2, nad1_pos2, nad4_pos2 | 3440-3711, 5051-6765, 1151-2079, 3713-5049 | GTR+I+G | 1419 |
|  | P6 | nad4l_pos3, nad1_pos3 | 3441-3711, 1152-2079 | GTR+G | 401 |
|  | P7 | nad2, nad6, nad3 | 2080-3090, 6766-7287, 3091-3438 | GTR+I+G | 627 |
|  | P8 | nad6_pos2, nad2_pos2, nad3_pos2 | 6767-7287, 2081-3090, 3092-3438 | GTR+I+G | 627 |
|  | P9 | nad2_pos3 | 2082-3090 | GTR+G | 337 |
|  | P10 | cox3_pos3, nad3_pos3 | 10269-11040, 3093-3438 | GTR+G | 374 |
|  | P11 | nad4_pos3 | 3714-5049 | GTR+G | 446 |
|  | P12 | nad5_pos3 | 5052-6765 | GTR+G | 572 |
|  | P13 | atp8, atp6_pos3, cox2_pos3, atp8_pos3, nad6_pos3 | 7957-8097, 7290-7956, 9615-10266, 7959-8097, 6768-7287 | GTR+I+G | 709 |
|  | P14 | atp8_pos2 | 7958-8097 | GTR+I+G | 47 |
|  | P15 | cox1, cox2 | 8098-9612, 9613-10266 | GTR+I+G | 723 |

**Table A2.** The best partitioning schemes and models for the Bayesian inference (BI) method based on PCG12 + 2 rRNAs + 22 tRNAs dataset selected by PartitionFinder.

| Dataset | Partitions | Partitioning scheme | Alignment | Best model | No. of sites | |
| --- | --- | --- | --- | --- | --- | --- |
| **PCG12 + 2 rRNAs + 22 tRNAs** | P1 | atp6, cytb, cox3 | 4859-5304, 1-766, 6845-7360 | GTR+I+G | 864 | |
|  | P2 | cox1_pos2, cox2_pos2, atp6_pos2, cytb_pos2, cox3_pos2 | 5400-6408, 6410-6844, 4860-5304, 2-766, 6846-7360 | GTR+I+G | 1587 | |
|  | P3 | nad4l, nad4, trnW, trnL2, trnP, trnM, trnH, trnQ, nad1, trnL1, nad5 | 2293-2474, 2475-3366, 10122-10188, 9547-9613, 9743-9806, 9614-9680, 9284-9345, 9807-9852, 767-1386, 9480-9546, 3367-4510 | GTR+I+G | 1859 | |
|  | P4 | nad4l_pos2, nad4_pos2, nad5_pos2, nad1_pos2 | 2294-2474, 2476-3366, 3368-4510, 768-1386 | GTR+I+G | 1419 | |
|  | P5 | nad6, nad2 | 4511-4858, 1387-2060 | GTR+I+G | 511 | |
|  | P6 | nad6_pos2, nad3_pos2, nad2_pos2 | 4512-4858, 2062-2292, 1388-2060 | GTR+I+G | 627 | |
|  | P7 | nad3, trnT, trnK, atp8_pos2, atp8 | 2061-2292, 10016-10079, 9410-9479, 5306-5398, 5305-5398 | GTR+I+G | 344 | |
|  | P8 | cox1, cox2 | 5399-6408, 6409-6844 | GTR+I+G | 723 | |
|  | P9 | rrnS, trnE, rrnL | 8213-8954, 9140-9175, 7361-8212 | GTR+G | 1630 | |
|  | P10 | trnY, trnS1, trnC, trnV, trnR, trnA, trnG, trnS2, trnD, trnN, trnI | 10189-10252, 9893-9950, 9017-9074, 10080-10121, 9853-9892, 8955-9016, 9240-9283, 9951-10015, 9075-9139, 9681-9742, 9346-9409 | GTR+I+G | 624 | |
|  | P11 | trnF | 9176-9239 | GTR+G | 64 | |
|  |  |  |  |  | |  |

**Table A3.** The best partitioning schemes and models for the Bayesian inference (BI) method based on PCG123 + 2 rRNAs + 22 tRNAs dataset selected by PartitionFinder.

| **Dataset** | **Partitions** | **Partitioning scheme** | **Alignment** | **Best model** | **No. of sites** |
| --- | --- | --- | --- | --- | --- |
| **PCG123 + 12 rRNA + 22 tRNA** | P1 | cox3, trnM, cytb, atp6 | 10267-11040, 13760-13826, 1-1149, 7288-7956 | GTR+I+G | 931 |
|  | P2 | cox1_pos2, cox2_pos2, atp6_pos2, cytb_pos2, cox3_pos2 | 8099-9612, 9614-10266, 7289-7956, 2-1149, 10268-11040 | GTR+I+G | 1587 |
|  | P3 | cox2_pos3, cytb_pos3 | 9615-10266, 3-1149 | GTR+G | 601 |
|  | P4 | nad4, nad4L, nad1, trnL1, nad5, trnQ, trnH | 3712-5049, 3439-3711, 1150-2079, 13626-13692, 5050-6765, 13953-14021, 13430-13491 | GTR+I+G | 1617 |
|  | P5 | nad4l_pos2, nad4_pos2, nad5_pos2, nad1_pos2 | 3440-3711, 3713-5049, 5051-6765, 1151-2079 | GTR+I+G | 1419 |
|  | P6 | nad4l_pos3, nad1_pos3 | 3441-3711, 1152-2079 | GTR+G | 401 |
|  | P7 | nad2, nad6 | 2080-3090, 6766-7287 | GTR+I+G | 511 |
|  | P8 | nad6_pos2, nad2_pos2, nad3_2 | 6767-7287, 2081-3090, 3092-3438 | GTR+I+G | 627 |
|  | P9 | nad2_pos3 | 2082-3090 | GTR+G | 337 |
|  | P10 | trnW, trnL2, trnP, trnV, nad3, trnT, trnK | 14332-14398, 13693-13759, 13889-13952, 14269-14331, 3091-3438, 14205-14268, 13556-13625 | GTR+G | 511 |
|  | P11 | atp8_pos3, nad6_pos3, atp6_pos3, cox3_pos3, nad3_pos3 | 7959-8097, 6768-7287, 7290-7956, 10269-11040, 3093-3438 | GTR+I+G | 818 |
|  | P12 | nad4_pos3 | 3714-5049 | GTR+G | 446 |
|  | P13 | nad5_pos3 | 5052-6765 | GTR+I+G | 572 |
|  | P14 | atp8_pos2, atp8, trnE, trnR, trnA | 7958-8097, 7957-8097, 13246-13299, 14022-14081, 13061-13122 | GTR+G | 270 |
|  | P15 | cox1, cox2 | 8098-9612, 9613-10266 | GTR+I+G | 723 |
|  | P16 | cox1_pos3 | 8100-9612 | GTR+G | 505 |
|  | P17 | rrnL, rrnS | 11041-12318, 12319-13060 | GTR+G | 2020 |
|  | P18 | trnD, trnI, trnN, trnS2, trnG, trnY, trnC, trnS1 | 13181-13245, 13492-13555, 13827-13888, 14140-14204, 13364-13429, 14399-14462, 13123-13180, 14082-14139 | GTR+I+G | 502 |
|  | P19 | trnF | 13300-13363 | GTR+G | 64 |
|  |  |  |  |  |  |

**Table A4.** The best partitioning schemes and models for the Bayesian inference (BI) method based on PCG123_AA dataset selected by PartitionFinder.

| **Dataset** | **Partitions** | **Partitioning scheme** | **Alignment** | Best model | **No. of sites** |
| --- | --- | --- | --- | --- | --- |
| **PCG123_AA** | P1 | atp6, cox3, cox2, cytb | 4162-4385, 5161-5397, 4937-5160, 1-381 | LG+I+G | 1066 |
|  | P2 | nad3. nad1, nad5 | 1675-2012, 382-689, 3434-4000 | LG+I+G | 1213 |
|  | P3 | nad4l, nad2 | 2013-2101, 690-1674 | LG+I+G | 1074 |
|  | P4 | nad4 | 2102-3433 | LG+I+G | 1332 |
|  | P5 | nad6, atp8 | 4001-4161, 4386-4434 | LG+I+G | 210 |
|  | P6 | cox1 | 4435-4936 | LG+I+G | 502 |
|  |  |  |  |  |  |

**Table A5.** The best partitioning schemes and models for the Bayesian inference (BI) method based on PCG123 + 2 rRNAs datasets selected by PartitionFinder.

| **Dataset** | **Partitions** | **Partitioning scheme** | **Alignment** | **Best model** | **No. of sites** |
| --- | --- | --- | --- | --- | --- |
| **PCG123 + 2 rRNA** | P1 | atp6, cytb, cox3 | 7288-7956, 1-1149, 10267-11040 | GTR+I+G | 864 |
|  | P2 | cox1_pos2, cox2_pos2, atp6_pos2, cox3_pos2, cytb_pos2 | 8099-9612, 9614-10266, 7289-7956, 10268-11040, 2-1149 | GTR+I+G | 1587 |
|  | P3 | cox1_pos3, cytb_pos3 | 8100-9612, 3-1149 | GTR+G | 888 |
|  | P4 | nad4, nad4l, nad1, nad5 | 3712-5049, 3439-3711, 1150-2079, 5050-6765 | GTR+I+G | 1419 |
|  | P5 | nad4l_pos2, nad4_pos2, nad5_pos2, nad1_pos2 | 3440-3711, 3713-5049, 5051-6765, 1151-2079 | GTR+I+G | 1419 |
|  | P6 | nad4l_pos3, nad1_pos3 | 3441-3711, 1152-2079 | GTR+G | 401 |
|  | P7 | nad2, nad6, nad3 | 2080-3090, 6766-7287, 3091-3438 | GTR+I+G | 627 |
|  | P8 | nad6_pos2, nad2_pos2, nad3_pos2 | 6767-7287, 2081-3090, 3092-3438 | GTR+I+G | 627 |
|  | P9 | nad2_pos3 | 2082-3090 | GTR+G | 337 |
|  | P10 | nad3_pos3, cox3_pos3 | 3093-3438, 10269-11040 | GTR+G | 374 |
|  | P11 | nad4_oos3 | 3714-5049 | GTR+G | 446 |
|  | P12 | nad5_pos3 | 5052-6765 | GTR+G | 572 |
|  | P13 | atp8_pos3, nad6_pos3 | 7959-8097 | GTR+I+G | 221 |
|  | P14 | cox2_pos3, atp8, atp6_pos3 | 9615-10266, 7957-8097, 7290-7956 | GTR+I+G | 488 |
|  | P15 | atp8_pos2 | 7958-8097 | GTR+I+G | 47 |
|  | P16 | cox1, cox2 | 8098-9612, 9613-10266 | GTR+I+G | 723 |
|  | P17 | rrnS, rrnL | 12319-13060, 11041-12318 | GTR+G | 2020 |

**Table A6.** The best partitioning schemes and models for maximum-likelihood (ML) analyses on PCG123 + 2 rRNAs + 22 tRNAs dataset selected by PartitionFinder.

| **Datasets** | Partitions | **Partitioning scheme** | **Best model** |
| --- | --- | --- | --- |
| **PCG123 + 2 rRNA + 22 tRNA** | P1 | cytb, atp6, cox3, trnK, trnM, trnT | GTR+F+I+G4 |
|  | P2 | cytb_pos2, atp6_pos2, cox1_pos2, cox2_pos2, cox3_pos2 | GTR+F+I+G4 |
|  | P3 | cytb_pos3 | GTR+F+G4 |
|  | P4 | nad1, nad4l, nad4, nad5 | GTR+F+I+G4 |
|  | P5 | nad1_pos2, nad4l_pos2, nad4_pos2, nad5_pos2 | GTR+F+I+G4 |
|  | P6 | nad1_pos3, nad4l_pos3 | GTR+F+G4 |
|  | P7 | nad2, nad3 | GTR+F+I+G4 |
|  | P8 | nad2_pos2, nad3_pos2, nad6_pos2 | GTR+F+I+G4 |
|  | P9 | nad2_pos3, nad6_pos3, atp8_pos3 | GTR+F+G4 |
|  | P10 | nad3_pos3, cox3_pos3 | GTR+F+I+G4 |
|  | P11 | nad4_pos3 | GTR+F+I+G4 |
|  | P12 | nad5_pos3 | GTR+F+I+G4 |
|  | P13 | nad6, atp8, atp8_pos2 | GTR+F+I+G4 |
|  | P14 | atp6_pos3, cox1_pos3, cox2_pos3 | HKY+F+G4 |
|  | P15 | cox1, cox2, trnQ | GTR+F+I+G4 |
|  | P16 | rrnL, rrnS, trnA, trnC, trnE, trnG | GTR+F+G4 |
|  | P17 | trnD, trnI, trnL1, trnL2, trnN, trnP, trnR, trnS1, trnV, trnW, trnY | GTR+F+I+G4 |
|  | P18 | trnF | GTR+F+I+G4 |
|  | P19 | trnH | GTR+F+I+G4 |
|  | P20 | trnS2 | GTR+F+I+G4 |

**Table A7.** The best partitioning schemes and models for maximum-likelihood (ML) analyses on PCG123 + 2 rRNAs dataset selected by PartitionFinder.

| **Dataset** | **Partitions** | **Partitioning scheme** | **Best model** |
| --- | --- | --- | --- |
| PCG123 + 2 rRNA | P1 | cytb, atp6, cox3 | GTR+F+I+I+R4 |
|  | P2 | cytb_pos2, atp6_pos2, cox1_pos2, cox2_pos2, cox3_pos2 | GTR+F+I+I+R3 |
|  | P3 | cytb_pos3, nad3_pos3, cox1_pos3 | TIM+F+R7 |
|  | P4 | nad1, nad4l, nad4, nad5 | TVM+F+R4 |
|  | P5 | nad1_pos2, nad4l_pos2, nad4_pos2, nad5_pos2 | GTR+F+I+I+R3 |
|  | P6 | nad1_pos3, nad4l_pos3 | TIM+F+I+I+R4 |
|  | P7 | nad2, nad3 | TIM2+F+I+I+R4 |
|  | P8 | nad2_pos2, nad3_pos2, nad6_pos2 | TVM+F+I+I+R3 |
|  | P9 | nad2_pos3, nad6_pos3, atp8_pos3 | GTR+F+R4 |
|  | P10 | nad4_pos3 | GTR+F+I+G4 |
|  | P11 | nad5_pos3 | GTR+F+I+G4 |
|  | P12 | nad6, atp8 | TIM2+F+I+G4 |
|  | P13 | atp6_pos3 | GTR+F+I+G4 |
|  | P14 | atp8_pos2 | GTR+F+I+G4 |
|  | P15 | cox1_pos2, cox2 | TIM2+F+I+G4 |
|  | P16 | cox2_pos3 | GTR+F+I+G4 |
|  | P17 | cox3_pos3 | GTR+F+I+G4 |
|  | P18 | rrnL, rrnS | GTR+F+R5 |

**Table A8.** The best partitioning schemes and models for maximum-likelihood (ML) analyses on PCG123 dataset selected by PartitionFinder.

| **Dataset** | **Partitions** | **Partitioning scheme** | **Best model** |
| --- | --- | --- | --- |
| PCG123 | P1 | cytb, cox3 | GTR+F+I+I+R5 |
|  | P2 | nad1 | GTR+F+I+G4 |
|  | P3 | nad2 | GTR+F+I+G4 |
|  | P4 | nad3 | GTR+F+I+G4 |
|  | P5 | nad4l | GTR+F+I+G4 |
|  | P6 | nad4 | GTR+F+I+G4 |
|  | P7 | nad5 | GTR+F+I+G4 |
|  | P8 | nad6 | GTR+F+I+G4 |
|  | P9 | atp6 | GTR+F+I+G4 |
|  | P10 | atp8 | GTR+F+I+G4 |
|  | P11 | cox1 | GTR+F+I+G4 |
|  | P12 | cox2 | GTR+F+I+G4 |
|  |  |  |  |

**Table A9.** The best partitioning schemes and models for maximum-likelihood (ML) analyses on PCG123_AA dataset selected by PartitionFinder.

| **Dataset** | Partitions | **Partitioning scheme** | **Best model** |
| --- | --- | --- | --- |
| PCG123_AA | P1 | cytb, nad4l, atp6, cox2, cox3 | mtMet+I+I+R4 |
|  | P2 | nad1, nad3, nad4, nad5 | mtMet+F+I+I+R5 |
|  | P3 | nad2 | LG+F+I+G4 |
|  | P4 | nad6, atp8 | mtMet+F+I+G4 |
|  | P5 | cox1 | LG+F+I+G4 |
|  |  |  |  |

**Table A10.** The best partitioning schemes and models for maximum-likelihood (ML) analyses on PCG12 + 2 rRNAs + 22 tRNAs datasets selected by PartitionFinder.

| **Dataset** | **Partitions** | **Partitioning scheme** | **Best model** |
| --- | --- | --- | --- |
| **PCG12 + 2 rRNA + 22 tRNA** | P1 | cytb, atp6, cox3 | GTR+F+I+G4 |
|  | P2 | nad1, nad4l, nad4, nad5, trnQ | TIM+F+I+G4 |
|  | P3 | nad2, trnP | TIM2+F+I+G4 |
|  | P4 | nad3, trnC, trnG | TIM+F+I+G4 |
|  | P5 | nad6 | GTR+F+I+G4 |
|  | P6 | atp8 | GTR+F+I+G4 |
|  | P7 | cox1, cox2 | TIM2+F+I+G4 |
|  | P8 | rrnL, rrnS, trnA, trnE | GTR+F+G4 |
|  | P9 | trnD, trnK, trnM, trnN, trnR, trnS1, trnT, trnV | GTR+F+I+G4 |
|  | P10 | trnF | GTR+F+I+G4 |
|  | P11 | trnH | GTR+F+I+G4 |
|  | P12 | trnI, trnL1, trnL2, trnS2, trnW, trnY | GTR+F+I+G4 |
|  |  |  |  |
